# An annotated cDNA library and microarray for large-scale gene-expression studies in the ant *Solenopsis invicta*

**DOI:** 10.1186/gb-2007-8-1-r9

**Published:** 2007-01-15

**Authors:** John Wang, Stephanie Jemielity, Paolo Uva, Yannick Wurm, Johannes Gräff, Laurent Keller

**Affiliations:** 1 Department of Ecology and Evolution, University of Lausanne, 1015 Lausanne, Switzerland; 2 Istituto di Ricerche di Biologia Molecolare, Merck Research Laboratories, 00040 Pomezia, Rome, Italy; 3 Brain Research Institute, University of Zürich/Swiss Federal Institute of Technology, 8057 Zürich, Switzerland

## Abstract

Ants display a range of fascinating behaviors, a remarkable level of intra-species phenotypic plasticity and many other interesting characteristics. Here we present a new tool to study the molecular mechanisms underlying these traits: a tentatively annotated expressed sequence tag (EST) resource for the fire ant *Solenopsis invicta*. From a normalized cDNA library we obtained 21,715 ESTs, which represent 11,864 putatively different transcripts with very diverse molecular functions. All ESTs were used to construct a cDNA microarray.

## Abstract 2

An annotated EST resource for the fire ant Solenopsis invicta containing 21,715 ESTs, which represent 11,864 putatively different transcripts, and a corresponding cDNA microarray are described.

## Background

Ants are important model species for sociobiology and behavioral ecology [1]. Life in an ant colony is marked by cooperation, but it also harbors conflicts. Both aspects have been studied extensively to understand the prerequisites for social behavior and to test the kin selection theory (reviewed in [2]). Other fascinating research areas in ants include self-organization, life-history evolution, as well as division of labor.

With the advent of new molecular and genomic techniques it is becoming possible to identify the genes underlying social behavior [3,4], as well as those involved in other interesting behaviors and traits. Unfortunately, in ants such studies have been seriously constrained by the lack of sequence data and other molecular tools. The majority of ant gene sequences have derived from two studies. A recent experiment examined differential gene expression in fire ants between winged virgin queens and wingless mated queens [5]. From this study 81 expressed sequence tags (ESTs) were submitted to GenBank. Another study, focusing on gene expression changes during the development of *Camponotus festinatus *workers, yielded 384 ESTs [6]. While informative, both of these studies were limited by the small number of genes examined. The goal of this project was, therefore, to create and sequence a much larger set of ant ESTs, namely for the ant *Solenopsis invicta*. Used in conjunction with DNA microarray technology [7,8], this sequence resource will allow us and other researchers to examine thousands of ant genes simultaneously.

*S. invicta *is one of the most extensively studied ant species. Also known as the red imported fire ant because of its accidental introduction to the United States from South America in the early 1900s and because of its painful, burning sting, this species has become a major agricultural and wildlife pest in the southern USA [9]. In attempts to control this species, its basic biology has been well elucidated [10,11]. Studies on *S. invicta *led the way in a number of research areas important for evolutionary biology: nest-mate conflicts over reproduction [12,13], sex-ratio conflicts [14,15], nepotism [16], chemical communication and warfare [17,18], and social evolution [19]. A particularly fascinating aspect of fire ant biology is that two distinct types of social organization exist in this species, and this is linked to a single gene, *Gp-9 *[20-22]. Colonies of the monogynous form are headed by a single reproductive queen with a specific *Gp-9 *genotype (*BB*), while colonies of the polygynous form contain up to several hundred reproductive queens that are all *Gp-9 *heterozygotes (*Bb*). The number of queens is regulated by workers, which will kill or tolerate additional queens based on their own and the queens' *Gp-9 *genotype [22]. This is one of a few cases where a complex social behavior is governed by a simple genetic mechanism.

We describe here a collection of 21,715 *S. invicta *ESTs generated from a normalized cDNA library. This library should encompass a maximum variety of genes, as it was derived from mRNA of all developmental stages of queens, males and workers from both colony types. Sequence assembly resulted in 11,864 putatively different genes. We have used a combination of blast analysis and protein pattern searches to obtain a preliminary Gene Ontology (GO) annotation for these genes. By comparison to the honey bee, we identified 23 potential Hymenoptera-specific genes. All ESTs were used to generate a high-density cDNA microarray, which will be a valuable resource for molecular, ecological and evolutionary studies in ants.

## Results and discussion

### Generation and assembly of fire ant ESTs

To survey the fire ant gene repertoire, we generated ESTs from a normalized cDNA library derived from ants of all developmental stages and castes (workers, queens, and males) of both the monogynous and polygynous social forms. First, we sequenced the 5' ends of 22,560 clones from the cDNA library. This yielded a total of 28,113 sequence reads, since about one-fourth of all clones were sequenced twice. From this set we then removed artifactual sequences and sequences smaller than 200 base pairs (bp; after vector and primer clipping), identifying 21,715 high-quality ESTs of 522 bp average length (Table 1).

To find redundant transcripts, the 21,715 ESTs were assembled into contiguous sequences (contigs, Table 1) using the Paracel Clustering Package. A total of 14,170 ESTs were assembled into 4,319 contigs, while the remaining 7,545 ESTs remained singleton sequences. In sum, there were 11,864 gene sets, hereafter referred to as assembled sequences, that putatively represent different transcripts. However, this number is expected to overestimate the true number of transcripts represented because some non-overlapping ESTs may represent the same gene and because assembly may have failed in case of alternative splicing, sequence polymorphism or sequencing errors. Assessed with a second independent method, the number of putatively different fire ant transcripts was indeed estimated at 'only' 9,770 (see below). The average length of all assembled sequences was 600 bp.

Since some of the cDNA clones were sequenced several times, 1,262 of the 4,319 contigs are due to re-sequencing, that is, composed of sequences of a single re-sequenced clone. The remaining 3,057 contigs are 'true contigs', that is, derived from at least two independent cDNA clones (Table 1).

### Quality of the cDNA clones and sequences

To obtain a tentative estimate of the percentage of 5' truncated transcripts, we compared the fire ant assembled sequences to a set of 3,951 proteins listed on the eukaryotic orthologous groups (KOG) database [23] that are highly conserved among *Drosophila melanogaster*, *Caenorhabditis elegans *and *Homo sapiens*. In total, 1,827 fire ant assembled sequences had a highly significant blastx hit (E ≤ 1e-20) to the *Drosophila *KOG proteins. Among these, 749 (41%) had regions of similarity that started within the 20 first amino-terminal amino acid residues of their *Drosophila *homologs with either an in-frame methionine at the same position as the fruitfly start methionine (588) or upstream of the alignment start (161). This suggests that up to 41% of the assembled sequences might have an intact 5' end, whereas the remaining 59% are probably 5' truncated.

The number of 3' truncated transcripts was harder to estimate because most cDNA clones (52.8%) were not sequenced all the way through to their 3' end (that is, the 5' sequence reads were shorter than most cDNA clones). Nevertheless, since 39.3% of all fire ant ESTs ended with a polyA sequence, up to 39.3% of our ESTs may have an intact 3' end. This is, however, likely to be an overestimate, as not all polyA sequences are true polyA tails.

Consistent with the expectation that the fire ant cDNA clones were sequenced from the 5' end, 92.2% of all assembled sequences with significant similarity to a gene in the non-redundant (nr) database were encoded on the plus strand. This estimate was obtained by counting how many times the open reading frames (ORFs) of the fire ant assembled sequences matched that of their best homologs in other organisms (see next section). However, a small percentage of the ant assembled sequences (7.8%) appeared to be encoded on the minus strand. This could be due to non-specific annealing of the SMART adaptors, to transcription of an adjacent gene pointing in the opposite orientation, or to the presence of antisense transcripts in our library.

To assess overall sequence quality, we computed the number of unresolved bases, marked as N by the base-calling program phred, present in all ESTs and assembled transcripts. The majority of sequences (83.7% of assembled sequences and 81.3% of all ESTs) had no unresolved bases. Another 15.8% of assembled sequences and 17.5% of ESTs had between one and three unresolved bases. Finally, a small percentage of sequences (0.5% of assembled transcripts and 1.2% of ESTs) had more than four unresolved bases.

### Comparative genomic analysis of fire ant cDNA data

**Figure 1:**
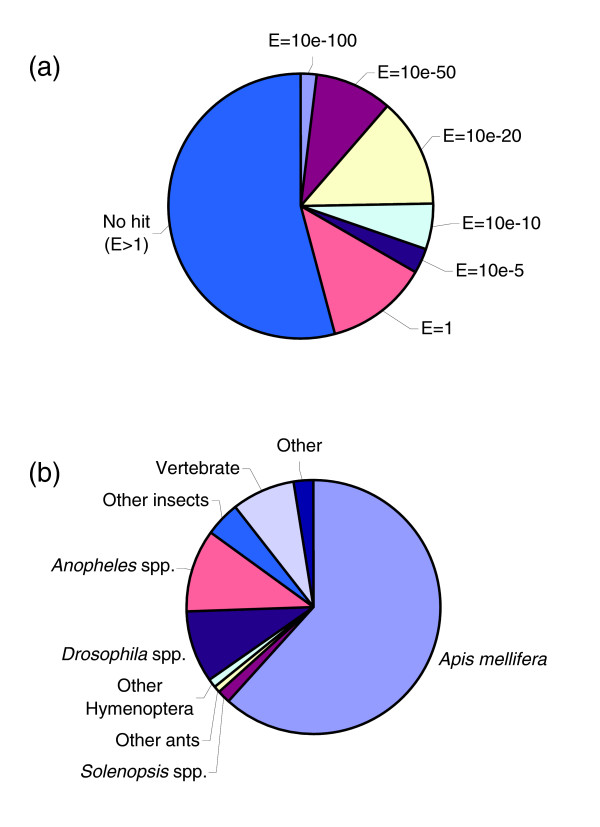
Sequence analysis by blastx searches. **(a) **Percentage of fire ant assembled sequences with and without blastx matches at various E-value cutoffs. **(b) **Quantitative overview of organisms providing the best-matching homologous protein sequences to fire ant assembled sequences (E ≤ 1e-5).

We used the blastx algorithm to compare the 11,864 fire ant assembled sequences to the nr database. Of these, 2,936 (24.7%) and 3,964 (33.4%) assembled sequences matched known or predicted protein-coding genes at a cutoff expectation value (E) of 1e-20 and 1e-5, respectively (Figure 1a). By contrast, 6,431 (54.2%) had no similarity at all to genes in the nr database (E > 1). For many of these 6,431 clones, the lack of detectible similarity may be because the sequenced region does not encompass a long enough ORF to meet the blastx comparisons' cutoff of 1. This may result from 5' truncation of cDNA clones (causing ESTs to consist mostly or entirely of 3' untranslated region), from a long 5' untranslated region, or from priming in intron regions of the pre-mRNAs. Alternatively, transcripts may lack large ORFs because they are short or because they are noncoding RNAs (that is, transcripts other than rRNA or tRNA that do not code for proteins). Noncoding RNAs are now thought to make up a considerable portion of the polyadenylated transcripts found in libraries such as ours [24,25]. For instance, in humans 57% of all polyadenylated transcripts might be noncoding RNAs [26].

Figure 1b depicts the 'best hit' for the 3,964 fire ant assembled sequences displaying significant similarity to known or predicted protein-coding genes. The best hit was a honey bee gene 61.6% of the time. This was expected, as the honey bee is the most closely related species with a fully sequenced genome. Due to the paucity of non-honey bee hymenopteran sequences in GenBank, for only 106 (2.7%) assembled sequences was the best hit a known ant gene; and only 41 (1.0%) assembled sequences were most related to a gene from hymenopteran species other than ants or the honey bee. An additional 953 (24.0%) fire ant assembled sequences were most similar to genes from non-hymenopteran insect species. Of these, 359 and 417 had best matches to fruitfly and mosquito genes, respectively. Interestingly, a subset of 320 genes (8.1%) shared their closest similarity with vertebrates, which is an observation that has also been made for the honey bee [27]. Other assembled sequences were most similar to genes from Nematoda (11) or other Animalia (26). Several had best matches to bacteria (4) or protozoa (13), possibly because these sequences were derived from microbes that infect fire ants or that have a commensal relationship with them. Alternatively, these sequences could be due to microbial contaminations acquired during sample collection. Finally, 17 assembled sequences appeared to be derived from viruses, including the recently identified *S. invicta *SINV-1 and SINV-1A viruses [28,29].

Interestingly, for 1,341 fire ant assembled sequences the best hit was a non-hymenopteran gene (bacterial, viral and protozoan hits excluded). This could be due to extensive sequence divergence between ant-bee gene pairs or gene loss in the bee. We examined these two alternatives using the recently completed and annotated honey bee genome sequence [30]. Most fire ant genes with a non-hymenopteran best hit (80.5%; 1,080/1,341) had a significant blastx hit to an annotated honey bee gene (Additional data file 1). Using tblastx, blastn or Ensembl (v38 Apr 2006 [31]) honey bee gene predictions, an additional 69 fire ant genes showed evidence for a potential honey bee homolog (Additional data file 1). Thus, for these 1,149 assembled sequences, sequence divergence is the likely reason for a non-hymenopteran best hit. Such sequence divergence could be due to directional selection in the honey bee lineage. The remaining 192 (14.3%) assembled sequences do not display significant similarity to the honey bee genome (Additional data file 1). This could be because some ant sequences are too short to meet the significance threshold for similarity (1e-5), extreme sequence divergence, or putative gene loss in the honey bee lineage.

We also used the blastx analysis described as an alternative method to estimate the number of unique fire ant genes sequenced. A total of 3,366 fire ant assembled sequences matched 2,772 different honey bee proteins, suggesting that 82.4% (2,772/3,366) of the fire ant assembled sequences may be unique. Thus, the 11,864 fire ant assembled sequences may represent 9,770 different genes. Assuming that the fire ant and the honey bee have a similar total number of genes (that is, 13,448 to 20,998 predicted genes, Ensembl v38 April 2006 [31]), this would represent approximately 46.5% to 72.7% of the genes in the fire ant genome.

In addition to the above-mentioned blastx searches to identify putative protein-coding genes, we carried out two other genomic analyses. First, to identify potential noncoding RNAs among the fire ant assembled sequences, we compared all assembled sequences via blastn to known noncoding RNAs from the NONCODE database [32] and the miRBase microRNA collection [33]. Consistent with the view that noncoding RNAs are often poorly conserved across taxa [25], the vast majority of fire ant sequences had no significant hits in these databases (E > 1e-5). Only one fire ant transcript (SiJWG03CAD.scf) was highly similar (E = 3e-14) to a known human microRNA (miRBase ID: hsa-mir-594). Second, we identified 772 assembled sequences conserved between the fire ant and the honey bee that fulfilled the following conditions: no resemblance to any known protein in the nr database (blastx, E > 1e-5), a good blastn hit against the honeybee genome (E ≤ 1e-5), and no significant blastn hit against other organisms (E > 1e-5). This list of genes (Additional data file 2) is likely to include transcripts with conserved untranslated region sequence motifs and some additional noncoding RNAs. However, it may also contain ant protein-coding genes that failed to have a blastx hit because they are truncated or because their honey bee homolog failed to be predicted during genome annotation.

### Functional annotation

Provisional functional annotation of the fire ant assembled sequences was done by adopting the GO annotation of the best-matching homologs in the nr database. At a blastx E-value cutoff of 1e-5, 3,964 fire ant assembled sequences displayed matches to proteins in the nr database. Of these, 3,035 (76.6%) could be annotated into at least one of the three main GO categories (biological process, molecular function, or cellular component) and 1,617 (40.8%) were in all three. The distribution of the fire ant assembled sequences among the main subcategories is summarized in Table 2 and the full GO assignments are in Additional data file 3. The most frequently identified molecular functions were 'binding' and 'catalytic activity' and those for biological process were 'physiological process' and 'cellular process' (Table 2). In addition to the annotation through blastx searches, GO classifications were assigned to fire ant assembled sequences based on the Prosite protein domains they contain (Table 2, Additional data file 4). These two GO annotations were then contrasted with the GO annotation of the *D. melanogaster *genome: The relative counts of fire ant genes were significantly different (hypergeometric distribution: p < 1e-8) from the relative counts of *Drosophila *genes in up to 23 second-level GO categories (Table 2). This could indicate that these gene categories are over- or underrepresented in the fire ant genome relative to the *Drosophila *genome. Alternatively, these gene categories may simply be biased in cDNA libraries relative to genomes, for instance, because they contain mainly highly or mainly lowly expressed genes. GO groupings and subcategories can be further explored using the AmiGO feature [34] of the Fourmidable database. As the annotations are automated, all functional assignments are tentative and considered at the 'inferred from electronic annotation' (IEA) level of evidence (see [35]).

### Being a Hymenopteran

The ants are classified within the order Hymenoptera, a group of insects including ants, bees and wasps. To identify Hymenoptera-specific genes, we looked for fire ant sequences that exhibited similarity only to genes from the honey bee or other Hymenoptera species. Using stringent criteria, we identified 148 fire ant sequences with strong similarity to the honey bee genome (tblastx, E < 1e-10) but no similarity to other known sequences (tblastx against non-hymenopteran sequences of the EMBL Nucleotide Sequence Database release 88; E > 1).

**Figure 2:**
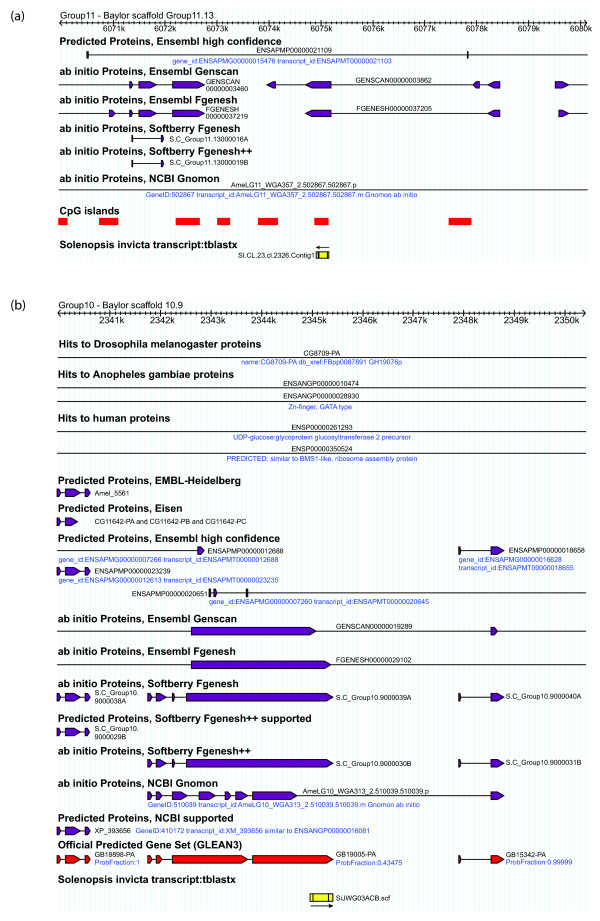
Examples of two candidate Hymenoptera-specific genes. **(a) **Fire ant sequence SI.CL.23.cl.2326.Contig1 matches an *ab intio *predicted honey bee gene that has no homology to any sequences in the public databases. The predicted gene was not included in the Honey Bee Official Gene Set. **(b) **Fire ant assembled sequence SiJWG03ACB.scf is the first EST evidence for the *ab initio *predicted honey bee gene GB19005-PA. Fire ant sequences are depicted as yellow boxes. Orientation (5' to 3') is indicated by an arrow. Predicted honey bee genes are depicted in purple; official Gene Set genes are shown in red. Images are based on output from Beebase (see Materials and methods).

As the fire ant sequences are not necessarily full-length, the region of ant-bee homology, while apparently Hymenoptera-specific, may be part of a larger and phylogenetically conserved protein. To investigate this possibility, we examined the surrounding honey bee genomic sequence (±5,000 bp) of each candidate Hymenoptera-specific gene. Genes predicted by homology with other organisms were found near most of our putative ant-bee pairs. These regions of ant-bee homology may simply be fragments of known genes that diverged in ants and bees. However, for 23 ant-bee gene pairs (Table 3, Figure 2, Additional data file 5), the predicted neighboring genes are either specific to bees or are transcribed in the opposite direction. Unless the region of ant-bee homology is part of a conserved gene with a large intron (that is, >5,000 bp), these 23 ant-bee gene pairs are strong candidate Hymenoptera-specific genes.

Further examination of these 23 candidate genes in hymenopteran species could prove interesting for understanding shared features. For instance, all Hymenoptera species have a haplodiploid sex determination system, with males developing from unfertilized haploid eggs and females from fertilized diploid eggs. Another feature found in many Hymenoptera is social behavior. Social behavior evolved independently in ants, bees and wasps [36,37] and, thus, it may be possible that a subset of the 23 ant-bee gene pairs was permissive for sociality to evolve or is important for social behavior.

### Behavior genes

To identify candidate genes that might be involved in the complex behavior of ants we compared the fire ant assembled sequences to a set of 106 *Drosophila *genes that are directly implicated in behavior [27]. Of these behavior genes, 17 (16%) matched at least one fire ant assembled sequence (Table 4). This value is less than the 44% (47/106; chi-squared, p < 5e-9) identified by the honey bee brain cDNA library [27], possibly because the honey bee cDNA library was specifically derived from brain tissue. We also compared the fire ant assembled sequences to all 636 *Drosophila *genes that had the GO annotation 'behavior'. Of these, 81 (13%) were good hits for at least 1 fire ant assembled sequence (Additional data file 6). In addition, some genes involved in complex behaviors in ants and other Hymenoptera may be specific to this taxon and not homologous to known genes.

### Viruses

In analyzing the cDNA library we noticed the presence of several viral transcripts. Seventeen fire ant assembled sequences were most similar to viral genes from RNA or DNA viruses (blastx, E < 1e-5; Table 5). Three sequences correspond to the recently identified SINV-1 virus, which possibly affects brood survival in *Solenopsis invicta *[28]. As the mutation rate in viruses can be high, we relaxed the E-value cutoff stringency to 1e-2, which yielded an additional nine putative viral genes. Based on different patterns of co-expression across several microarray experiments (unpublished data) the 26 putative viral genes could represent at least 5 different viruses.

To verify that these ESTs are from fire ant viruses and not from viruses infecting the insects fed to the ants, we tried to re-amplify all putative viral ESTs from fire ant cDNA derived from eggs, larvae and pupae. Out of 26 ESTs, 15 amplified when using egg and/or pupal cDNA as a template. Since eggs and pupae do not eat and either lack an intestine or have emptied their intestine, these 15 ESTs most likely stem from genuine fire ant viruses. Another five ESTs, including the three SINV-1 ESTs, amplified only in ant larvae. For these larvae-specific ESTs and the remaining six ESTs that amplified in none of the cDNA categories tested, additional tests would be needed to verify that they stem from fire ant viruses.

Further characterization of viruses in fire ants may be useful for two main reasons. First, as fire ants are an invasive pest species that causes considerable economic damage in the southern USA and other locations, viruses have been suggested as possible agents of fire ant control. Second, viruses can have dramatic effects on the behavior of their hosts. For instance, the Kakugo virus has been suggested to increase the aggressiveness of honey bee workers, as infected workers are much more likely to defend the nest against hornets than non-infected nestmates [38]. Another virus is most likely involved in superparasitism behavior in the parasitoid wasp *Leptopilina boulardi *[39]. It would be interesting to determine if the viruses identified by our EST project manipulate fire ant behavior to promote viral transmission or if they could be used for fire ant control.

### Longevity

Ant queens and workers show up to ten-fold lifespan differences, although they develop from the same eggs and are thus genetically identical [1]. Lifespan differences must, therefore, stem from differences in gene expression, making ants a useful system to study aging and lifespan determination [40,41]. The average lifespan of fire ant queens is estimated at six to seven years [42], while workers are thought to have an average lifespan of ten to 70 weeks [1]. We have identified fire ant homologs (blastx, E < 1e-20) to several genes that are likely involved in determining the lifespan of invertebrate model organisms (reviewed in [43,44]): Cu-Zn superoxide dismutase (SI.CL.3.cl.379.Contig1), Mn superoxide dismutase (SI.CL.16.cl.1663.Contig1), catalase (SI.CL.40.cl.4085.Contig1), histone deacetylase Rpd3 (SiJWG06ABE.scf), Indy (SI.CL.40.cl.4047.Contig1) and the heatshock transcription factor HSF-1 (SiJWH04BCB2.scf). It will be exciting to test whether these homologs are expressed at different levels in the long-lived queens and the short-lived workers. In addition, comparing fire ant queens to fire ant workers using functional genomic approaches may help identify new candidate aging genes.

### Highly expressed genes

In total, 67 contigs contained more than 10 ESTs (Additional data file 7). Consistent with the hypothesis that these are highly expressed genes, we found several homologs to ribosomal genes and other housekeeping genes in this subset. The largest contig (SI.CL.0.cl.071.Contig1) contained 48 clones. Based on blastx searches this gene encodes a small (74 amino acid residue) protein of unknown function. Interestingly, this gene is highly conserved across vertebrates, arthropods and fungi. For instance, the putative fire ant protein and its zebra fish homolog share 79% amino acid residues. While the majority of the 67 highly expressed transcripts had significant blastx matches to well-characterized proteins, 18 (26.9%) did not match any known sequence (E > 1e-5 for both blastx and blastn).

### Fire ant microarray

To permit functional genomic analysis for the fire ant we produced a cDNA microarray using all 22,560 clones sequenced from the cDNA library. We successfully PCR-amplified 17,685 (78.4%) cDNAs (only one strong band, Additional data file 8), which putatively represent 10,122 (85.3%) of the fire ant assembled sequences (Additional data file 9). To evaluate the percentage of cDNA spots derived from legitimate and sufficiently highly expressed transcripts, we examined the signal-to-background ratio of all spots in four test hybridizations (for details and additional analysis see Additional data files 10, 11 and 12). The two samples compared were derived from a mix of adults (workers, virgin queens, and males from both colony types in equal amounts) and a mix of brood (eggs, larvae and pupae of all castes in equal amounts). Of the spots derived from a single good PCR product, 82.8% (14,642/17,685) had an interpretable signal (that is, signal intensity greater than background plus two standard deviations), indicating that most cDNA clones are derived from legitimate transcripts.

### Future prospects

The extraordinary complexity and diversity of morphology, behavior, and social organization in ants is far from being understood from a molecular genetics point of view. The present work, the largest collection of ESTs for an ant species, provides a valuable sequence, clone, and genomic resource for the ant research community. Using this resource it will be possible to identify genes important in caste determination, behavioral genetics and plasticity, chemical communication, and population control. This microarray should also allow comparisons across related species. More broadly, as the genome sequence for the social honey bee, *Apis mellifera*, is available and that for the solitary wasp, *Nasonia vitripennis*, will soon arrive, comparisons and contrasts of both gene sequence and expression among the three species might shed light onto hymenopteran biology, behavior and social organization.

## Conclusion

We have sequenced 22,560 ESTs from a normalized fire ant cDNA library and assembled them into 11,864 putatively unique transcripts. Using comparative genomic analyses and the GO vocabulary, we have functionally annotated the fire ant ESTs into a broad range of molecular functions and biological processes. Examination of the fire ant genes has led to the identification of 23 putative Hymenoptera-specific genes. Finally, we have developed a cDNA microarray that will be useful for large-scale gene expression profiling.

## Materials and methods

### Ants

Monogynous and polygynous fire ant colonies were collected in Georgia (USA) in 2003 and 2004 and transferred to the laboratory as previously described [45]. Colonies were maintained in climate-controlled rooms at 25°C and fed with crickets, mealworms, a mix of vegetables, and a mix of canned tuna fish, dog food and peanut butter. Samples were collected manually and immediately frozen in liquid nitrogen.

### cDNA library

Using the Trizol reagent (Invitrogen, Carlsbad, CA, USA), total RNA was isolated from various samples of both monogynous and polygynous nests: eggs, small larvae, medium-sized larvae, sexual larvae, as well as pupae and adults of males, workers and queens (including both virgin and mated queens). We then pooled about 1 μg of each RNA sample to create a master sample with a maximum diversity of transcripts. This master sample was precipitated once with LiCl to eliminate contaminating DNA, quality checked on a 1% agarose gel and a Bioanalyzer 2100 chip (Agilent, Santa Clara, CA, USA) and sent in ethanol to Evrogen (Moscow, Russia) for cDNA library construction.

Evrogen constructed a normalized cDNA library using the SMART technology, which should enrich for full-length sequences. The plasmid used was pAL16. Based on PCR amplification of the inserts of 2,300 clones, the mean and median cDNA clone length was estimated at 940 bp and 850 bp, respectively. The shortest cDNA clone from this subset measured 180 bp, while the longest one measured about 3,300 bp. By comparison, the average *Drosophila *cDNA clone was 2 kb and the longest clone was 8.7 kb [46], suggesting that the fire ant cDNA library has many short clones that do not represent the entire transcriptional unit. Although the fire ant cDNA library is not directional, a 2 bp difference between the 3' and 5' SMART adaptors on all inserts permits sequencing cDNA clones specifically from the 5' end.

### Sequencing and sequence analysis

For 22,560 clones selected at random from the cDNA library, approximately 600 bp-sequence reads were obtained from the insert 5' end. Of these clones, 5,573 were sequenced in duplicate (mostly both times from the 5' end, with the exception of 77 clones that were sequenced from both the 3' and the 5' end). The primer used for the first approximately 8,000 sequences was SMART tag2 5'-AAGCAGTGGTA**T**CAACGCAGAGTACG-3' (which forms a 1 bp mismatch, in bold); the primer used for all other sequences was SMART tag2 fixed 5'-AAGCAGTGGTAACAACGCAGAGTACG-3' (which matches perfectly). Sequencing was done by Synergene (Schlieren, Switzerland) on plasmid DNA extracted from overnight cultures. Base calling was performed with phred [47,48]. The Paracel Clustering Package (Paracel, Pasadena, CA, USA) was used to filter low-quality sequences (base calls with phred values <15 and EST length <200 bp), to remove vector and SMART adaptor sequence, as well as to mask polyA tails and other repetitive sequences. In addition, Paracel was used to identify and assemble redundant transcripts: ESTs that had an overlap of >50 bp were, when possible, automatically assembled into contiguous sequences (contigs). ESTs that did not meet this criterion were called singletons.

In order to find homologs of the fire ant assembled sequences in other organisms, all singletons and contigs were used to interrogate public sequence databases. Blast sequence alignments [49,50] were performed using the Blast Network Service provided by the Swiss Institute for Bioinformatics or on a desktop PC using standalone blast software. For both blastx and blastn searches the default settings were used. E-values are reported at 1e-5, except where indicated otherwise.

### Gene Ontology annotation

We used the blastx algorithm to compare all 11,864 assembled sequences against the nr protein database. Using the best GO annotated SwissProt or TrEmbl hit with an E-value ≤ 1e-5, we annotated our transcripts at the IEA evidence level. Additionally, we scanned all assembled sequences for Prosite patterns with the stand-alone ps_scan perl program using the default cutoff level of 0 [51]. Transcripts having a Prosite pattern with a GO annotation were also annotated with the same GO terms at the IEA evidence level. In order to compare the fire ant GO annotations to those of *D. melanogaster*, we downloaded the *D. melanogaster *genome GO annotation from [52] on 19 September 2006. The WEGO web tool [53] was used to calculate the relative numbers of second-level GO categories within each first-level GO category (molecular function, biological process, cellular component) for both species. Using the hypergeometric test in R, we then tested which GO categories were significantly over- or underrepresented in the fire ant cDNA library relative to the *Drosophila *genome. Bonferroni correction was applied to the 80 tests carried out to correct for multiple comparisons.

### Fourmidable database

A MySQL database with web interface was produced to house the fire ant EST and assembled sequence data (P Uva *et al*., manuscript in preparation). Users can view sequence trace files, perform blast searches against fire ant assembled sequences, download sequences, browse through blastx and GO annotations, and so on. The database is publicly accessible [54].

### Identification of Hymenoptera-specific genes

All fire ant assembled sequences were compared against the nr protein database via blastx. The 6,948 transcripts that did not show strong similarity to the non-hymenopteran sequences of the nr database (blastx using BLOSUM45; E > 1) were subsequently aligned to the honey bee genome (build Amel 4.0). Of these, 216 ant transcripts had strong similarity to honey bee sequences (tblastx using BLOSUM45; E ≤ 1e-10). These 216 sequences were compared against all non-honey bee sequences of the EMBL Nucleotide Sequence Database (release 88, September 2006). We retained the 148 ant transcripts that showed strong similarity to honey bee build 4.0 (E ≤ 1e-10) and no or very weak similarity (E > 1) to known non-hymenopteran sequences (tblastx using BLOSUM45). When multiple tblastx alignment frames were possible, the positive strand frame with the strongest E-value was retained. The 10,000 bp honey bee genomic region surrounding each ant-bee sequence pair was then compared against the nr protein database via blastx. For 31 ant transcripts, the corresponding honey bee genomic region either did not show similarity to known genes, or only showed similarity to genes transcribed in the opposite direction. InterProScan was used to scan for protein signatures [55]. Additionally, the ant transcripts were aligned via tblastx against build 2.0 of the honey bee genome, which is currently the bee genome version with the most extensive annotation. With these results a GFF annotation file was generated and uploaded to BeeBase [56] for visual examination of all ant transcript-honey bee genome homolog pairs. Based on the existence and orientation of surrounding predicted genes we then determined a confidence level for each ant-bee pair. We assigned three stars when an ant transcript overlapped with a previously known bee gene (*ab initio *prediction or EST evidence); two stars if there was no known bee gene close by; one star if a gene from another organism appeared to hit within 5,000 bp of the ant-bee pair. In addition, 8 ant-bee pairs considered as false positives were eliminated, leaving us with 23 candidate Hymenoptera-specific genes. BeeBase was used to generate Additional data file 5 and a preliminary version of Figure 2, which was subsequently reformatted and modified to contain only relevant data: redundant text was removed, non-empty tracks were collapsed and empty tracks were deleted.

### Microarray construction

Bacteria clones were inoculated into PCR plates containing 5 μl modified LB-ampicillin broth (0.2 × LB without NaCl) and grown overnight. Plasmid inserts were amplified by PCR after adding 95 μl of PCR mix. A single primer, SMART PCR primer 5'-AAGCAGTGGTAACAACGCAGAGT-3', which matches both the 3' and 5' SMART adaptor of the inserts, was used. PCR mixes contained 0.4 μl 5 U/μl TAQ (Qiagen, Hilden, Germany), 10 μl 10 × Qiagen buffer, 20 μl Q solution, 4 μl 25 mM MgCl_2_, 1.5 μl 25 mM dNTPs, and 1 μl 100 μM SMART PCR primer. An initial 9 minute denaturation at 94°C was followed by 40 cycles of 30 s at 94°C, 30 s at 59°C, and 3 minutes at 72°C. The reaction ended with an additional incubation of 7 minutes at 72°C. PCR products (2 μl of each) were analyzed on a 1% agarose gel. Gel pictures were visually examined to classify all PCR products as follows: 'strong single band' (78.4%); 'no band' (3.9%); or 'weak or multiple bands' (17.5%). These data were used to create an Excel file (Additional data file 8), which will allow microarray users to exclude data from non-single-band spots. We preferred this solution to printing only single-band PCR products, as this would have involved an error-prone rearraying step.

PCR products were purified by a standard NaOAc/ethanol precipitation, resuspended in 30 μl water and transferred into duplicate 384-well plates using a Biomek FX liquid-handling robot (Beckman Coulter, Fullerton, CA, USA). Then PCR products were dried and resuspended in 20 μl 3 × SSC, 1.5 M betaine. This spotting buffer improves spot homogeneity and signal-to-noise ratio [57]. We also resuspended 48 times 10 commercial exogenous controls (SpotReport Alien cDNA Array Validation System, Stratagene, La Jolla, CA, USA) in 3 × SSC, 1.5 M betaine, 1 set for each subgrid of the microarray. Microarrays were printed on aldehydesilane-coated slides (NexterionTM Slide AL, Schott Nexterion, Jena, Germany), using an OmniGrid 300 spotting robot (GeneMachines, San Carlos, CA, USA). Spot and printing quality were assessed visually under a dissecting microscope after printing. While a few slides had minor defects (for example, a few spots missing or damaged by dust particles), the majority of slides exhibited no defects. DNA was crosslinked to slides by baking at 80°C for 1 h. Afterwards, the slides were post-processed with NaBH_4 _using the manufacturer's recommended protocol.

### Clone tracking

To detect major mistakes (for example, inverted or rotated plates) made during sequencing, amplification and/or transfer into 384-well plates, we resampled and sequenced 534 PCR products from the 384-well plates. These samples were chosen so that they represented 2 to 4 samples of each 96-well plate. For all 96-well plates we also manually checked that PCR length patterns corresponded roughly to sequence length patterns. Using these 2 quality control methods, we identified 8 96-well plates that had been sequenced upside-down. After careful verification involving more sequencing, we corrected these mistakes by renaming the sequences correctly. At that point only 6 control sequences (1.1%) did not match the expected sequence, suggesting that these were sporadic contaminations.

### Availability of sequence data, cDNA clones and microarrays

The ESTs described in this paper were submitted to the GenBank data library under accession numbers EE127747 to EE149461. The assembled sequences can be downloaded from the Fourmidable database [54]. The microarray data were submitted to Gene Expression Omnibus [58] with accession number GSE5995. Fire ant cDNA clones and cDNA microarrays can be obtained according to instructions on Fourmidable [54].

## Additional data files

The following additional data are available with the online version of this paper. Additional data file 1 lists honey bee sequences similar to fire ant assembled sequences with a non-honey bee best hit. Additional data file 2 lists all fire ant assembled transcripts with a significant blastn hit to the honey bee genome and no other blastx or blastn hit. Additional data file 3 shows the GO annotations for all assembled transcripts based on blastx searches. Additional data file 4 shows the GO annotations for all assembled transcripts based on Prosite searches. Additional data file 5 shows the honey bee genome regions surrounding the candidate Hymenoptera-specific genes listed in Table 3. Additional data file 6 contains fire ant assembled sequences similar to *D. melanogaster *genes with the GO term 'behavior'. Additional data file 7 contains an annotated list of the most abundant transcripts. Additional data file 8 shows the PCR results for the cDNA clones deposited onto the microarray. Additional data file 9 shows which fire ant assembled sequences had at least one cDNA clone with a good (single-band) PCR product. Additional data file 10 gives details on the microarray analyses performed. Additional data file 11 lists the fire ant clones that are differentially expressed between adults and brood based on a 4-fold cutoff. Additional data file 12 lists the fire ant clones that are differentially expressed between adults and brood based on a *t*-test (p < 0.001).

## Supplementary Material

Additional data file 1 Honey bee sequences similar to fire ant assembled sequences with a non-honey bee best hit Click here for file

Additional data file 2 All fire ant assembled transcripts with a significant blastn hit to the honey bee genome and no other blastx or blastn hit Click here for file

Additional data file 3 GO annotations for all assembled transcripts based on blastx searches Click here for file

Additional data file 4 GO annotations for all assembled transcripts based on Prosite searches Click here for file

Additional data file 5 Honey bee genome regions surrounding the candidate Hymenoptera-specific genes listed in Table 3 Click here for file

Additional data file 6 Fire ant assembled sequences similar to *D. melanogaster *genes with the GO term 'behavior' Click here for file

Additional data file 7 Annotated list of the most abundant transcripts Click here for file

Additional data file 8 PCR results for the cDNA clones deposited onto the microarray Click here for file

Additional data file 9 Fire ant assembled sequences that had at least one cDNA clone with a good (single-band) PCR product Click here for file

Additional data file 10 Details on the microarray analyses performed Click here for file

Additional data file 11 Fire ant clones that are differentially expressed between adults and brood based on a 4-fold cutoff Click here for file

Additional data file 12 Fire ant clones that are differentially expressed between adults and brood based on a *t*-test (p < 0.001) Click here for file

## Figures and Tables

**Table 1:** Fire ant EST and assembly statistics

Total number of sequence reads	28,133
cDNA clones sequenced from 5' end	22,560
Extra reads due to re-sequencing	5,573
High-quality sequences after filtering*	21,715
Average EST size after trimming (bp)	522.4
Total number of assembled sequences	11,864
Number of contigs	4,319
True contigs (from >2 different clones)	3,057
Re-sequencing contigs^†^	1,262
Number of singletons	7,545
Number of putatively different fire ant sequences	<11,864
Average size of assembled sequences (bp)	600.5

*High quality sequences are those with greater than 200 bp after trimming of vector and primer sequences and with a phred value higher than 15. In addition, this set excludes artifactual sequences that were manually removed. ^†^Contigs composed of replicate sequences of only one clone

**Table 2:** Gene Ontology annotation

	***Solenopsis invicta *EST library**						***D. melanogaster *genome**
		
	**Blastx-determined GO**			**Prosite-determined GO**			
**Molecular function**	4,301*		(100.0%)	486*		(100.0%)	14,778* (100.0%)
Antioxidant activity	20		(0.5%)	2		(0.4%)	39 (0.3%)
Binding	**1,765**	↑	**(41.0%)**	174		(35.8%)	4,319 (29.2%)
Catalytic activity	**1,456**	↑	**(33.9%)**	**201**	↑	**(41.4%)**	4,072 (27.6%)
Chaperone regulator activity	**5**	↑	**(0.1%)**	0		(0.0%)	1 (0.0%)
Enzyme regulator activity	91		(2.1%)	7		(1.4%)	382 (2.6%)
Molecular function unknown	**145**	↓	**(3.4%)**	**6**	↓	**(1.2%)**	1,852 (12.5%)
Motor activity	29		(0.7%)	1		(0.2%)	88 (0.6%)
Nutrient reservoir activity	**14**	↑	**(0.3%)**	0		(0.0%)	8 (0.1%)
Obsolete molecular function	0		(0.0%)	**9**	↑	**(1.9%)**	0 (0.0%)
Signal transducer activity	**153**	↓	**(3.6%)**	**4**	↓	**(0.8%)**	1,091 (7.4%)
Structural molecule activity	210		(4.9%)	59		(12.1%)	759 (5.1%)
Transcription regulator activity	**116**	↓	**(2.7%)**	4		(0.8%)	841 (5.7%)
Translation regulator activity	**62**	↑	**(1.4%)**	7		(1.4%)	92 (0.6%)
Transporter activity	235		(5.5%)	12		(2.5%)	1,014 (6.9%)
Triplet codon-amino acid adaptor activity	**0**	↓	**(0.0%)**	0		(0.0%)	220 (1.5%)
**Cellular component**	4,838*		(100.0%)	362*		(100.0%)	14,986* (100.0%)
Cell^†^	**1,868**	↑	**(38.6%)**	147		(40.6%)	5,225 (34.9%)
Cellular component unknown	**85**	↓	**(1.8%)**	**0**	↓	**(0.0%)**	1,920 (12.8%)
Envelope	107		(2.2%)	1		(0.3%)	290 (1.9%)
Extracellular matrix	14		(0.3%)	0		(0.0%)	46 (0.3%)
Extracellular matrix part	4		(0.1%)	0		(0.0%)	23 (0.2%)
Extracellular region	**73**	↓	**(1.5%)**	2		(0.6%)	416 (2.8%)
Extracellular region part	23		(0.5%)	0		(0.0%)	88 (0.6%)
Membrane-enclosed lumen	160		(3.3%)	3		(0.8%)	515 (3.4%)
Organelle	**1,360**	↑	**(28.1%)**	100		(27.6%)	3,007 (20.1%)
Organelle part	548		(11.3%)	22		(6.1%)	1,632 (10.9%)
Protein complex	575		(11.9%)	**87**	↑	**(24.0%)**	1,756 (11.7%)
Synapse	7		(0.1%)	0		(0.0%)	40 (0.3%)
Synapse part	3		(0.1%)	0		(0.0%)	27 (0.2%)
Virion^†^	**11**	↑	**(0.2%)**	0		(0.0%)	1 (0.0%)
**Biological process**	5,453*		(100.0%)	630*		(100.0%)	22,798* (100.0%)
Biological process unknown	**61**	↓	**(1.1%)**	**0**	↓	**(0.0%)**	888 (3.9%)
Cellular process	**2,242**	↑	**(41.1%)**	**297**	↑	**(47.1%)**	7,772 (34.1%)
Development	**121**	↓	**(2.2%)**	**0**	↓	**(0.0%)**	2,148 (9.4%)
Growth	17		(0.3%)	0		(0.0%)	102 (0.4%)
Interaction between organisms	6		(0.1%)	0		(0.0%)	92 (0.4%)
Physiological process	**2,328**	↑	**(42.7%)**	**315**	↑	**(50.0%)**	7,858 (34.5%)
Pigmentation	1		(0.0%)	0		(0.0%)	51 (0.2%)
Regulation of biological process	436		(8.0%)	11		(1.7%)	1,658 (7.3%)
Reproduction	**18**	↓	**(0.3%)**	**0**	↓	**(0.0%)**	826 (3.6%)
Response to stimulus	**207**	↓	**(3.8%)**	7		(1.1%)	1,402 (6.1%)
Viral life cycle	**16**	↑	**(0.3%)**	0		(0.0%)	1 (0.0%)

Listed are the numbers and percentages of assembled fire ant sequences and of *D. melanogaster *genes that match at least one of the second-level GO terms for molecular function, cellular component, or biological process. GO annotations for fire ant sequences were inferred electronically using two methods: blastx homology to GO-annotated proteins and Prosite protein domain scans. Statistically significant over- (↑) or underrepresentation (↓) of GO terms in fire ant relative to the *Drosophila *genome are indicated in bold (p < 10^-8^, Bonferroni-corrected hypergeometric test). *This number represents the sum of the numbers of occurences of GO terms below this level. ^†^The 'cell part' and 'virion part' GO categories were excluded from analyses because they were redundant with the 'cell' and 'virion' categories, respectively.

**Table 3:** Putative Hymenoptera-specific genes

	*Solenopsis invicta *assembled sequence^1^					Blast statistics		*Apis mellifera *sequence						Confidence^7^
		
Identifier (length)	Span	Frame	ORF^2 ^(bp)	I^3^	Exp^4^	Bit-score	E-value	Linkage Group	Span	Strand	ORF^2 ^(bp)	Est^5^	Annotated gene^6^	
SI.CL.8.cl.881.Contig1 (724 bp)	509-640	2	300		•	272	1.24E-18	6	2701427-2701558	+	429		*Ab initio *prediction	***

SI.CL.8.cl.843.SiJWH04BDO2.scf (730 bp)	582-761	3	147		•	210	1.99E-12	NW_001254419.^8^	44307-44486	-	147	•	Near NH homology. GB18184-PA on reverse strand	**

SI.CL.19.cl.1938.Contig1 (835 bp)	21-323	3	372	T	•	212	1.43E-12	6	1145090-1145392	-	429		*Ab initio *prediction. Near GB12791-PA on reverse strand	***

SI.CL.19.cl.1953.SiJWC11BBX.scf (613 bp)	81-215	3	555		•	166	5.08E-08	8	5253595-5253729	-	372	•	GB14543-PA. Near NH homology on reverse strand	*
								
	306-416					87	4.5E-15		5252894-5253094		306			
								
	435-635					200			5253189-5253299		318			

SI.CL.23.cl.2326.Contig1 (632 bp)	413-577	2	219		•	291	1.33E-20	11	8022183-8022347	+	480		*Ab initio *prediction	***

SI.CL.26.cl.2688.Contig1 (859 bp)	60-131	3^9^	87		•	98	9.74E-15	9	10421877-10421948	-	549	•	*Ab initio *prediction. Near NH homology on reverse strand	**
						
	119-256	2^9^	558			186			10421751-10421888					

SI.CL.33.cl.3311.Contig1 (710 bp)	228-359	3	189		•	258	3.07E-17	14	8634060-8634191	-	132	•	Near *ab initio *prediction. Near NH homology on reverse strand	*

SI.CL.33.cl.3384.Contig1 (469 bp)	229-327	1^9^	264	T,S	•	160	3.11E-13	14	3770768-3770866	-	231		*Ab initio *prediction	***
								
	362-454	2^9^	180	S		104			3770649-3770741		186			

SI.CL.35.cl.3595.Contig1 (415 bp)	123-398	3	342		•	301	5.97E-22	NW_001261806.^8^	12471-12746	+	327		*Ab initio *prediction	***

SiJWA02BAZ2.scf (600 bp)	374-469	2	261		•	193	2.13E-15	5	9909503-9909598	+	627	•	Near GB15931-PA and NH homology on reverse strand	*
						
	533-604					98			9909356-9909427					

SiJWA03CAW.scf (666 bp)	49-144	1	96			120	2.1E-16	NW_001259848.^8^	47860-47955	+	99	•	GB10007-PA on reverse strand	***
										
	136-297		117			182			47704-47865		726			

SiJWA12ACK.scf (212 bp)	137-268	2^9^	69		•	264	1.42E-19	3	5151467-5151598	+	162	•	Near *ab initio *prediction and NH homology on reverse strand	**
								
	63-143	3^9^	72			69			5151391-5151471		189			

SiJWB12BCQ.tag5_B12_04.scf (754 bp)	121-369	1	354		•	254	1.1E-16	7	5620128-5620376	+	336		*Ab initio *prediction on reverse strand	***

SiJWC11BAT.scf (342 bp)	189-278	3	228		•	160	3.98E-17	14	8645843-8645932	+	162	•	Near *ab initio *prediction and homology	**
								
	282-368					123	6.41E-14		8645754-8645840		117			

SiJWE02BBO2.scf (865 bp)	714-863	3	129		•	243	1.26E-15	6	4850974-4851123	-	354		Near *ab initio *prediction on reverse strand	**

SiJWF07BCC.tag5_F07_11.scf (799 bp)	329-529	2	96		•	196	6.59E-11	3	6205208-6205408	-	108		Near NH homology. *Ab initio *prediction on reverse strand	**

SiJWG01BDU2.scf (759 bp)	21-227	3	102		•	354	1.23E-26	2	9618145-9618351	+	171	•	GB12576-PA and NH homology on reverse strand	*

SiJWG03ACB.scf (623 bp)	172-609	1	471		•	558	4.63E-47	10	2344965-2345402	+	1440	•	GB19005-PA	***

SiJWH02AAN.scf (469 bp)	100-294	1	102		•	341	1.32E-30	12	281374-281568	-	294		-	***
										
	28-105		69			104			281564-281641		207			

SiJWH05BDPR5A08.scf (658 bp)	580-657	1	78		•	161	1.1E-15	10	2890267-2890344	+	159	•	Near *ab initio *prediction	**

SiJWH05BDV2.scf (517 bp)	204-353	3	198		•	237	4.87E-15	5	6704423-6704572	+	174		*Ab initio *prediction	**

SiJWH08AAT.scf (653 bp)	76-162	1	60		•	141	4.53E-20	5	1169177-1169263	+	84	•	Near *ab initio *prediction and NH homology	*
										
	151-195		102			75	4.52E-13		1169261-1169305		69			

SiJWH08ADY.scf (563 bp)	236-496	2	327		•	312	1.32E-22	12	4477772-4478032	-	432		GB16574-PA	***

^1^*Solenopsis invicta *assembled sequences that show no significant similarity to any known non-hymenopteran sequence (E > 1), but high similarity to a region of the honey bee genome (E < e-10). ^2^Length in base-pairs of the largest overlapping in-frame open reading frame. ^3^In-frame Interproscan annotation of fire ant assembled sequence. T means 'transmembrane region', S means 'signal peptide'. ^4^Gene is known (•) to be expressed in fire ant (unpublished microarray data). ^5^In honey bee, EST evidence exists (•) within 5,000 bp of the aligned region. ^6^This column shows the annotation of overlapping or nearby (within 5,000 bp) honey bee genes, as well as the nearby presence of genes from non-hymenopteran organisms. Numbers starting with GB are honeybee Official Gene Set numbers. '*Ab initio *prediction' indicates that Gnomon, Genscan, or another algorithm was used to predict a gene that was not retained for the bee genome Official Gene Set. 'NH homology' indicates the nearby presence of a gene from non-hymenopteran organisms. ^7^Based on visual inspection we assigned a confidence level (the more asterisks the better) to each ant-bee putative gene pair (see Materials and methods). ^8^*Apis mellifera *unanchored scaffolds such as NW_001254419.1 are regions that have not been mapped to a chromosome. ^9^Multiple alignment frames for a *S. invicta *transcript indicate possible frameshifts during sequencing.

**Table 4:** Fire ant assembled sequences putatively involved in behavior

Fire ant assembled sequence	*Drosophila *polypeptide ID	Gene name and behavior in *Drosophila*	E-value
SI.CL.10.cl.1087.Contig1	CG5670-PB	*Na pump alpha subunit*	1.0e-134
SI.CL.13.cl.1344.SiJWC08BDJ.scf	CG4443-PA	*courtless *(courtship behavior)	1.0e-73
SI.CL.13.cl.1344.Contig1	CG4443-PA	*courtless *(courtship behavior)	5.0e-73
SiJWE02ABO.scf	CG3263-PG	*cAMP-dependent protein kinase R1 *(olfactory learning)	4.0e-66
SiJWA12BCM.scf	CG2212-PA	*swiss cheese*	1.0e-65
SiJWC02AAC2.scf	CG3966-PA	*neither inactivation nor afterpotential A*	3.0e-55
SiJWB06ABV.scf	CG4379-PB	*cAMP-dependent protein kinase 1 *(locomotor rhythm, memory, olfactory learning and rhythmic behavior)	2.0e-42
SI.CL.3.cl.316.Contig1	CG8472-PB	*calmodulin*	2.0e-42
SI.CL.20.cl.2069.Contig1	CG2212-PB	*swiss cheese*	5.0e-42
SiJWH05AEA.scf	CG2048-PC	*discs overgrown *(altered behavioral response to cocaine)	4.0e-40
SiJWH06BAG.scf	CG8472-PB	*calmodulin*	4.0e-39
SI.CL.9.cl.956.Contig1	CG14724-PB	*cytochrome c oxidase subunit Va*	6.0e-38
SiJWA04BDS2.scf	CG3331-PA	*ebony *(locomotor rhythm)	7.0e-38
SiJWG01ADR.scf	CG7826-PC	*minibrain *(circadian rhythm and olfactory learning)	1.0e-24
SiJWD02ACW.scf	CG7758-PA	*pumpless*	1.0e-24
SI.CL.31.cl.3101.Contig1	CG1232-PB	*temperature-induced paralytic E*	3.0e-16
SiJWG06BCF2.scf	CG5670-PA	*Na pump alpha subunit*	8.0e-15
SiJWF02BDZ.scf	CG32688-PA	*hyperkinetic *(flight behavior)	1.0e-13
SiJWB11ABH.scf	CG10033-PG	foraging*	1.0e-11
SiJWB03ACL.scf	CG7100-PH	*cadherin-N*	2.0e-11
SiJWD03ACB.scf	CG10697-PA	*aromatic-L-amino-acid decarboxylase *(courtship behavior and learning and/or memory)	1.0e-07

*Although the best hit for SiJWB11ABH.scf is *foraging*, a type I cGMP-dependent protein kinase (PKG), when using blastx analysis with only the *Drosophila *predicted proteins, closer inspection using all the nr sequences suggests that it is actually a type II PKG.

**Table 5:** Fire ant assembled sequences most similar to viral genes

Fire ant assembled sequence	Best virus hit ID	Hit description	E-value	Identity (%)
SI.CL.23.cl.2338.Contig1	Q5Y974	Structural polyprotein. [*Solenopsis invicta *virus 1]	0	98
SI.CL.23.cl.2338.Contig2	Q5Y974	Structural polyprotein. [*Solenopsis invicta *virus 1]	0	92
SI.CL.8.cl.873.Contig1	Q65353	ORF B. [*Autographa californica *nuclear polyhedrosis virus]	2.0e-76	52
SiJWG09BAM.scf	Q5Y975	Nonstructural polyprotein. [*Solenopsis invicta *virus 1]	2.0e-63	96
SiJWF01ADQ.scf	Q6AW71	(orf1)RNA-dependent RNA polymerase. [*Bombyx mori *Macula-like latent virus]	3.0e-51	93
SiJWB11ACS.scf	Q6AW71	(orf1)RNA-dependent RNA polymerase. [*Bombyx mori *Macula-like latent virus]	1.0e-44	90
SI.CL.29.cl.2930.Contig1	Q65353	ORF B. [*Autographa californica *nuclear polyhedrosis virus]	1.0e-43	55
SI.CL.28.cl.2823.Contig1	Q38QJ4	Polyprotein. [Kelp fly virus]	7.0e-34	28
SiJWC03CAP.scf	Q5ZNV0	Hypothetical protein. [*Cotesia congregata *bracovirus]	2.0e-22	51
SiJWA06BBH.scf	Q85431	RNA polymerase. [Rice stripe virus]	1.0e-21	35
SI.CL.37.cl.3723.Contig1	Q5S8C7	Non-structural polyprotein (Fragment). [Honey bee virus - Israel]	1.0e-18	40
SI.CL.41.cl.4135.Contig1	Q38QJ4	Polyprotein. [Kelp fly virus]	2.0e-15	34
SI.CL.19.cl.1909.Contig1	Q6AW70	(orf2)Coat protein. [*Bombyx mori *Macula-like latent virus]	2.0e-14	84
SI.CL.6.cl.610.Contig1	Q8QY61	Polyprotein. [Sacbrood virus]	2.0e-11	26
SI.CL.25.cl.2511.Contig1	O11437	(pv4)Non-capsid protein. [*Urochloa *hoja blanca virus]	6.0e-11	26
SI.CL.6.cl.610.Contig3	Q9QRA8	Polyprotein (Fragment). [Tomato ringspot virus]	2.0e-10	23
SI.CL.6.cl.610.Contig2	Q3YC01	Polyprotein (Fragment). [Stocky prune virus]	2.0e-06	29
SiJWA06CAM.scf	Q6QLR4	(RdRp)RNA-dependent RNA polymerase (Fragment). [*Venturia canescens *picorna-like virus]	3.0e-05	37
SiJWC05ADI.scf	Q5ZP67	Soluble protein. [*Cotesia congregata *bracovirus]	7.0e-05	38
SI.CL.40.cl.4005.Contig1	P03515	(N)Nucleocapsid protein (Nucleoprotein). [*Punta toro *phlebovirus]	4.0e-04	32
SiJWG01BBJ2.scf	Q9JGN8	(p1vc)P1. 339K. [Rice grassy stunt virus]	0.001	23
SiJWD07ACK.scf	Q8BDE0	Replicase polyprotein. [Acute bee paralysis virus]	0.002	25
SI.CL.10.cl.1089.Contig1	Q9YMJ7	Envelope protein. [*Lymantria dispar *multicapsid nuclear polyhedrosis virus]	0.003	23
SI.CL.16.cl.1675.Contig1	Q9YW13	(MSV079)Hypothetical protein MSV079. [*Melanoplus sanguinipes *entomopoxvirus]	0.004	42
SiJWH05ADG.scf	Q76LW4	Polyprotein. [Kakugo virus]	0.008	27
SiJWE11AAZ.scf	Q5ZNU9	Soluble protein. [*Cotesia congregata *bracovirus]	0.01	34
