# Supplementary material for: An annotated cDNA library and microarray for large-scale gene-expression studies in the ant Solenopsis invicta
Source: Genome Biol. 2007 Jan 15;8(1):R9. doi: 10.1186/gb-2007-8-1-r9 (PMC1839134; doi:10.1186/gb-2007-8-1-r9)

**Additional Data File 5: Putative Hymenoptera-specific Genes**
The 10,000 bp region around the ant-bee alignment of every putative Hymenoptera-specific gene is depicted relative to the annotated honey bee genome.

The honey bee genome assembly 2.0 chromosome coordinates are at the top of each image. Below, the alignment of proteins from *A. gambiae, C. elegans, D. melanogaster, H. sapiens* and *S. cerevisiae* are shown. Additionally, honey bee proteins predicted by several different groups (Eisen, EMBL, Ensembl, NCBI) as well as with multiple *ab initio* prediction methods are displayed. Finally, genes from the honey bee Official Gene Set (GLEAN3) are in red. The alignment of fire ant transcripts are in light blue; 5' to 3' direction is indicated by an arrow.

Coordinates may differ from those in Table 3 because Table 3 was generated using assembly 4 of the honey bee genome.

Index of subfigures:


| | page |
|---|---|
| a. SiJWA02BAZ2.scf | 2 |
| b. SiJWA03CAW.scf | 3 |
| c. SiJWA12ACK.scf | 4 |
| d. SiJWB12BCQ.tag5_B12_04.scf | 5 |
| e. SiJWC11BAT.scf | 6 |
| f. SI.CL.8.cl.881.Contig1 | 7 |
| g. SI.CL.8.cl.843.SiJWH04BDO2.scf | 8 |
| h. SI.CL.19.cl.1938.Contig1 | 9 |
| i. SI.CL.19.cl.1953.SiJWC11BBX.scf | 10 |
| j. SI.CL.23.cl.2326.Contig1 | 11 |
| k. SI.CL.26.cl.2688.Contig1 | 12 |
| l. SI.CL.33.cl.3311.Contig1 | 13 |
| m. SI.CL.33.cl.3384.Contig1 | 14 |
| n. SI.CL.35.cl.3595.Contig1 | 15 |
| o. SiJWE02BBO2.scf | 16 |
| p. SiJWF07BCC.tag5_F07_11.scf | 17 |
| q. SiJWG01BDU2.scf | 18 |
| r. SiJWG03ACB.scf | 19 |
| s. SiJWH02AAN.scf | 20 |
| t. SiJWH05BDPR5A08.scf | 21 |
| u. SiJWH05BDV2.scf | 22 |
| v. SiJWH08AAT.scf | 23 |
| w. SiJWH08ADY | 24 |

**a.SiJWA02BAZ2.scf**

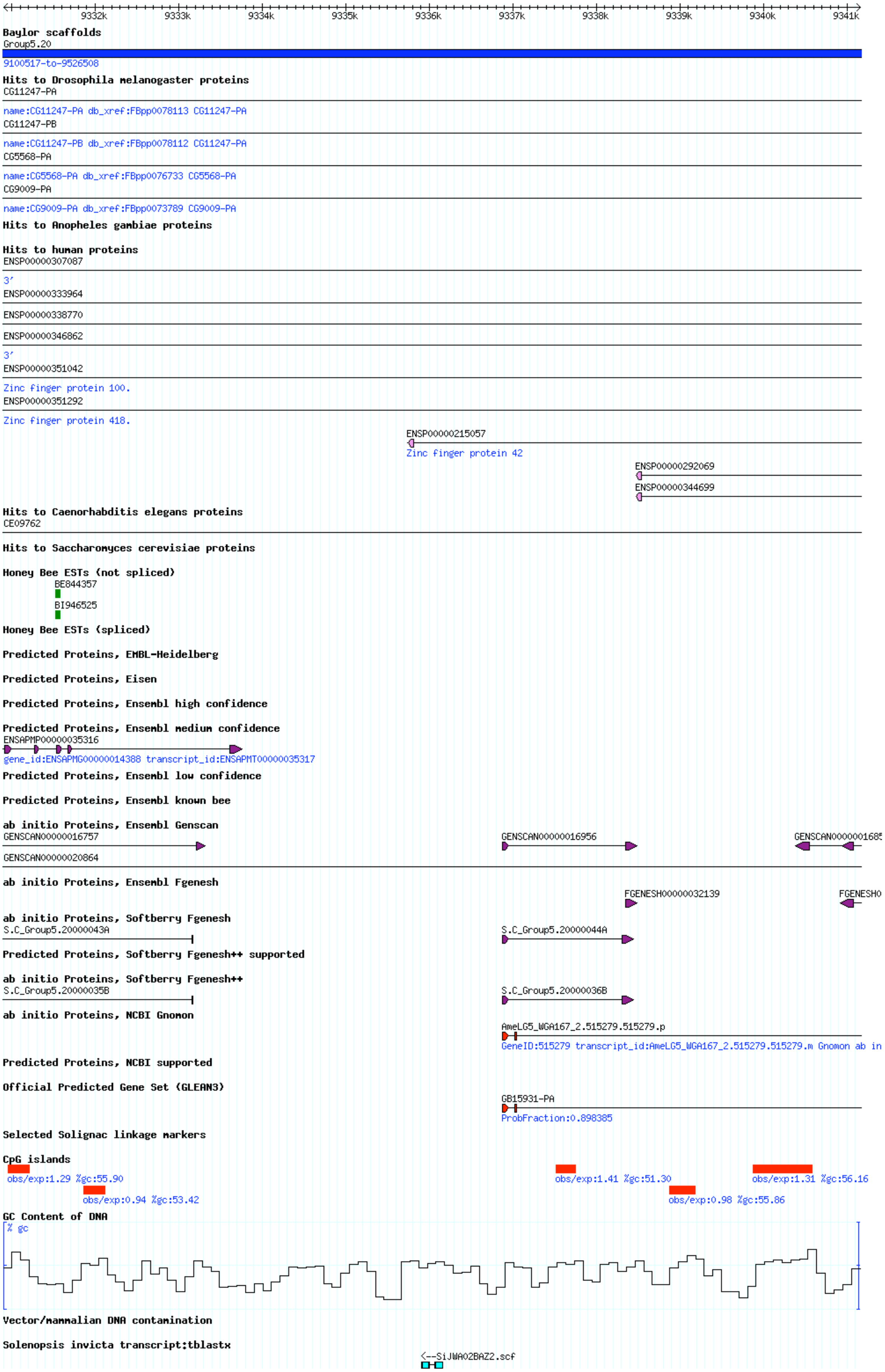

## b.SiJWA03CAW.scf

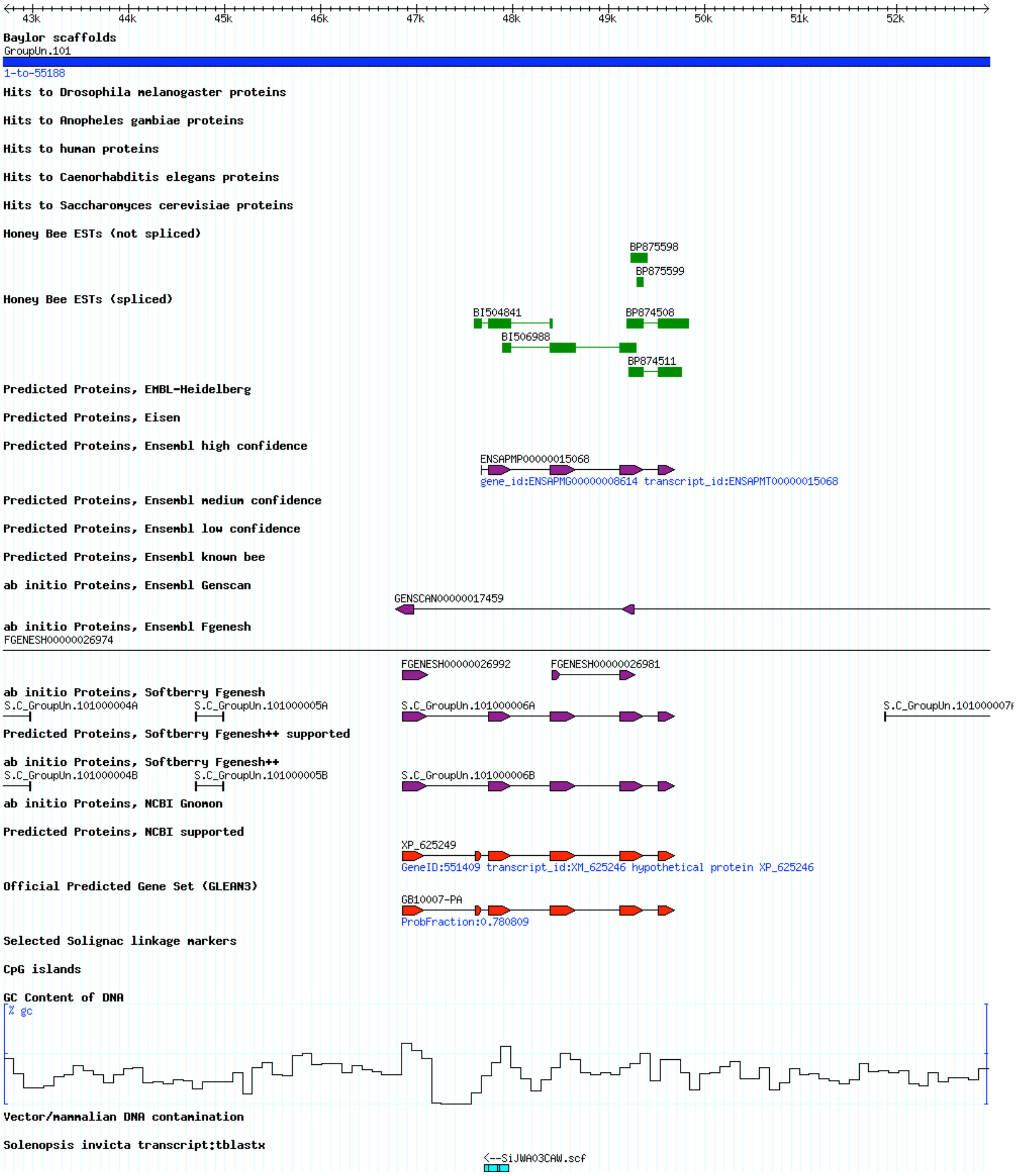

# c.SiJWA12ACK.scf

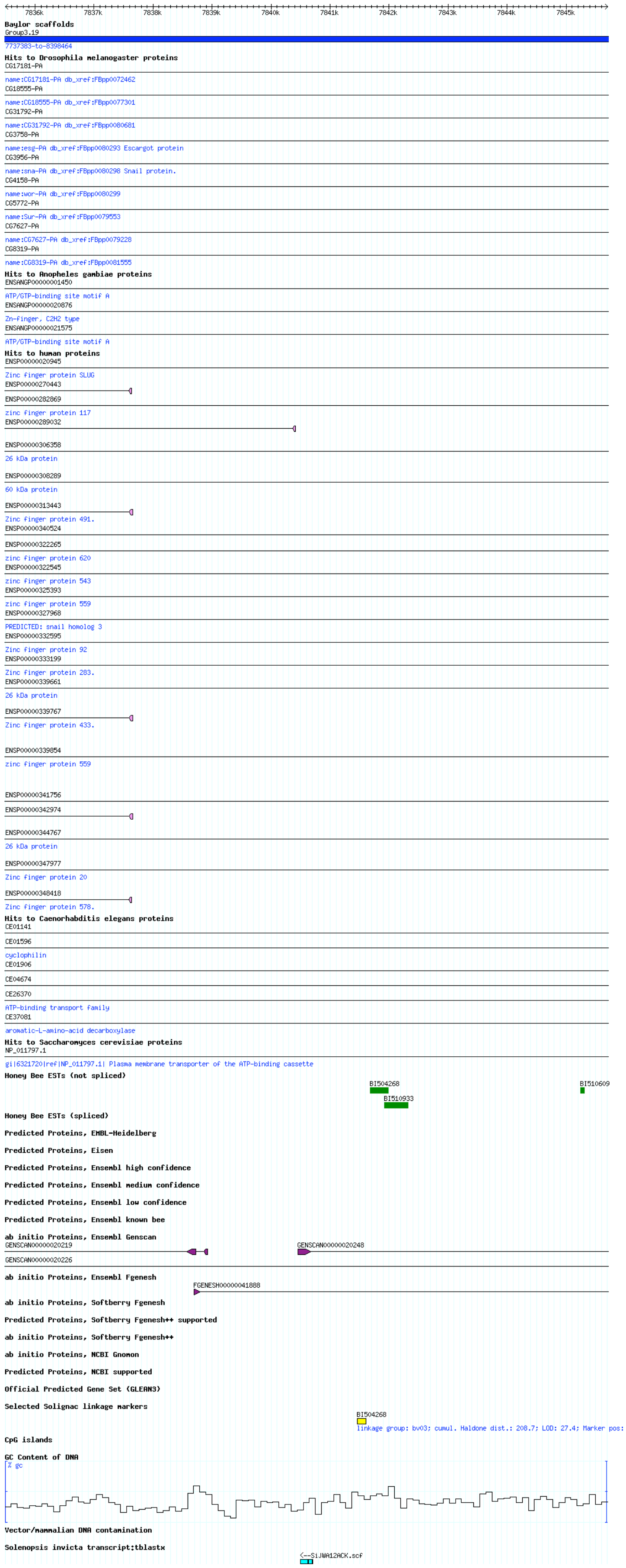

# d.SiJWB12BCQ.tag5_B12_04.scf

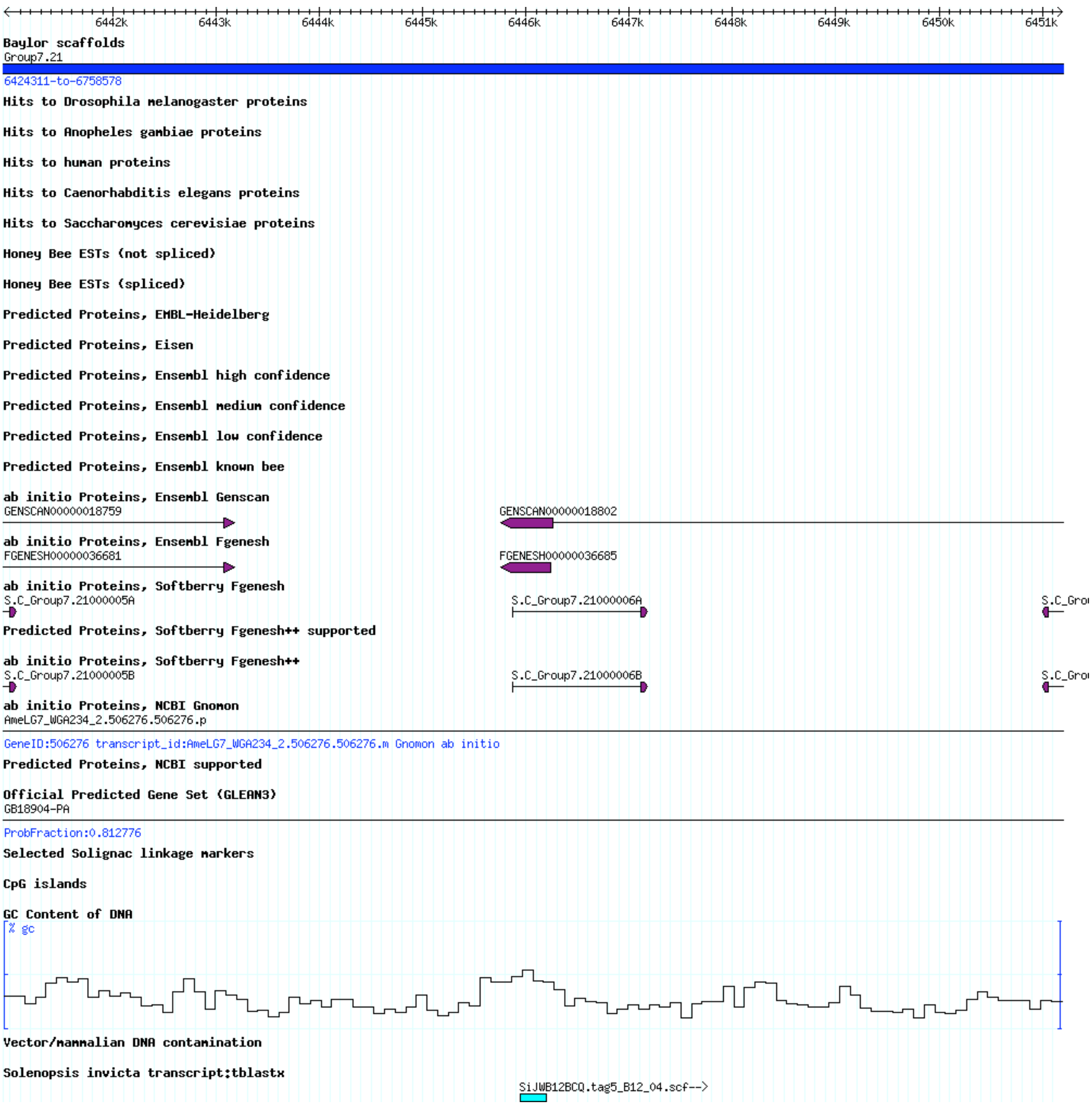

# e.SiJWC11BAT.scf

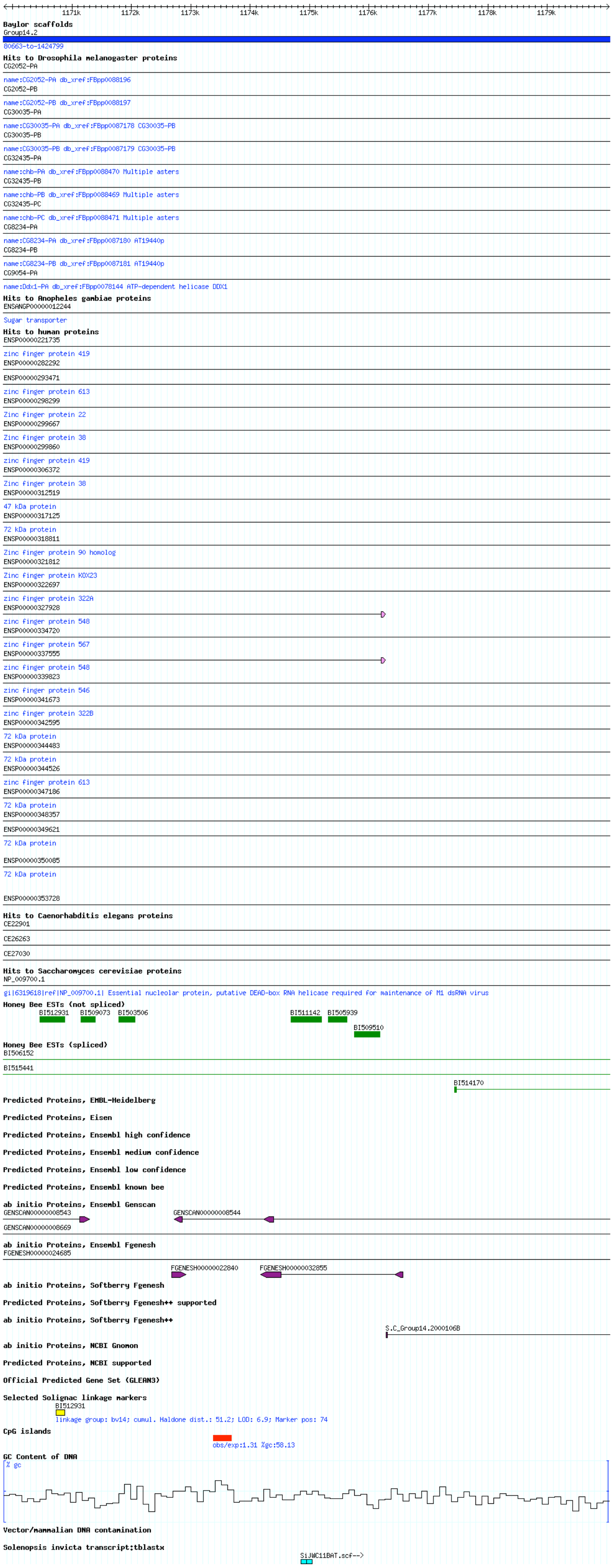

## f. SI.CL.8.cl.881.Contig1

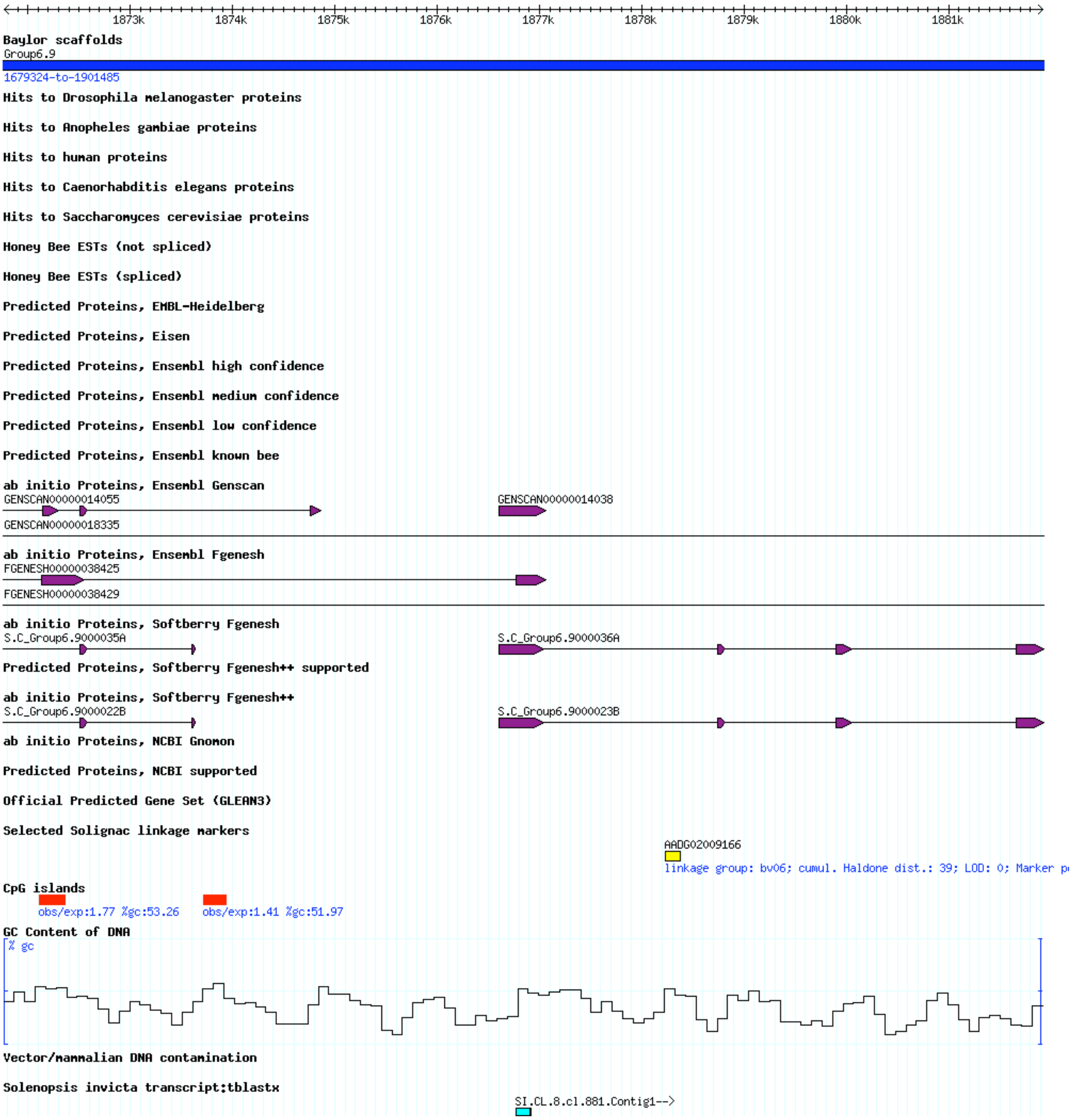

# g.SI.CL.8.cl.843.SiJWH04BDO2.scf

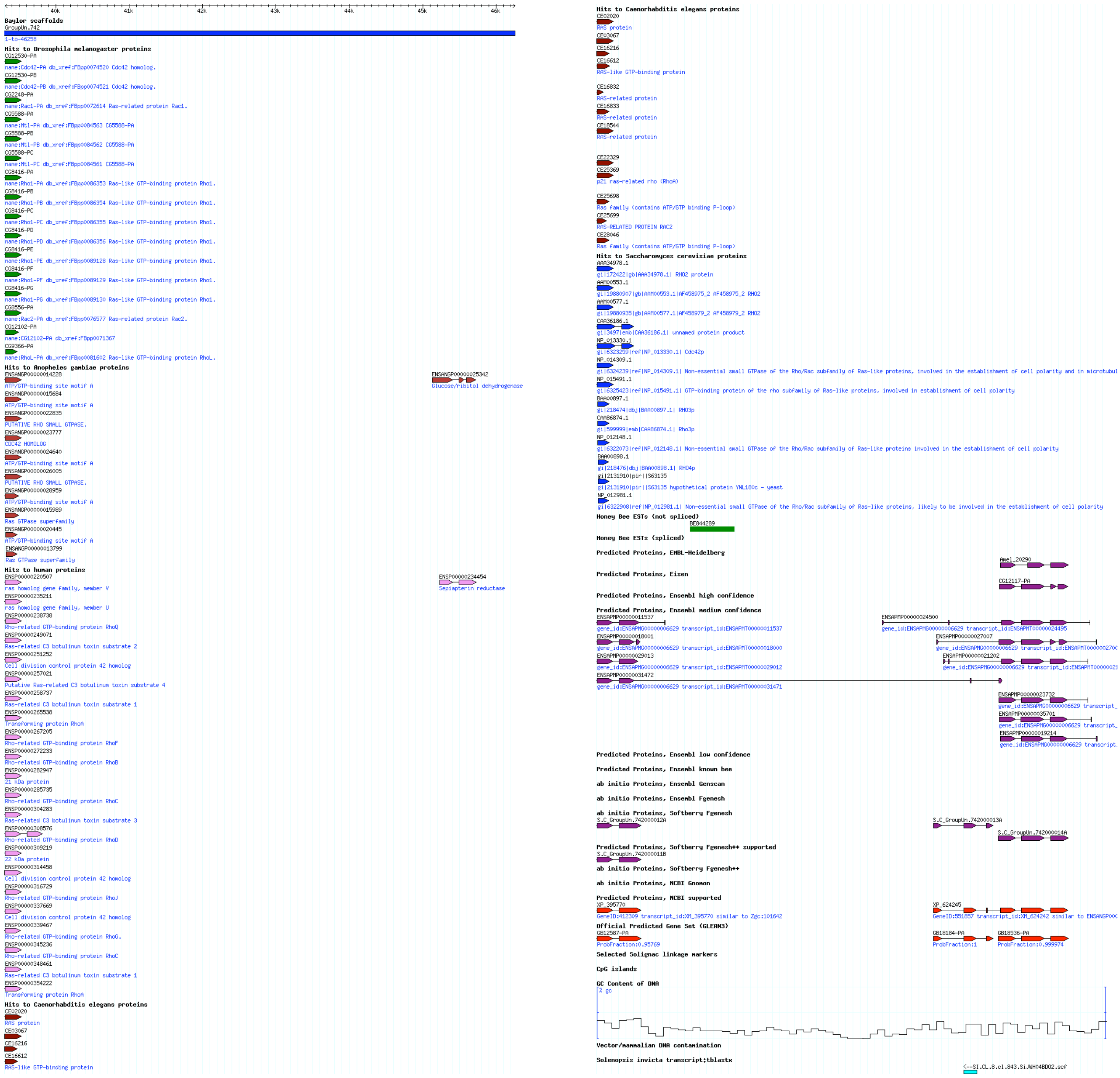

* SI.CL.8.cl.843.SiJWH04BDO2.scf aligns close to one extreme end of the unanchored genomic scaffold GroupUn.742, which is why there is only little annotation data to the right side of SI.CL.8.cl.843.SiJWH04BDO2.scf.

## h. SI.CL.19.cl.1938.Contig1

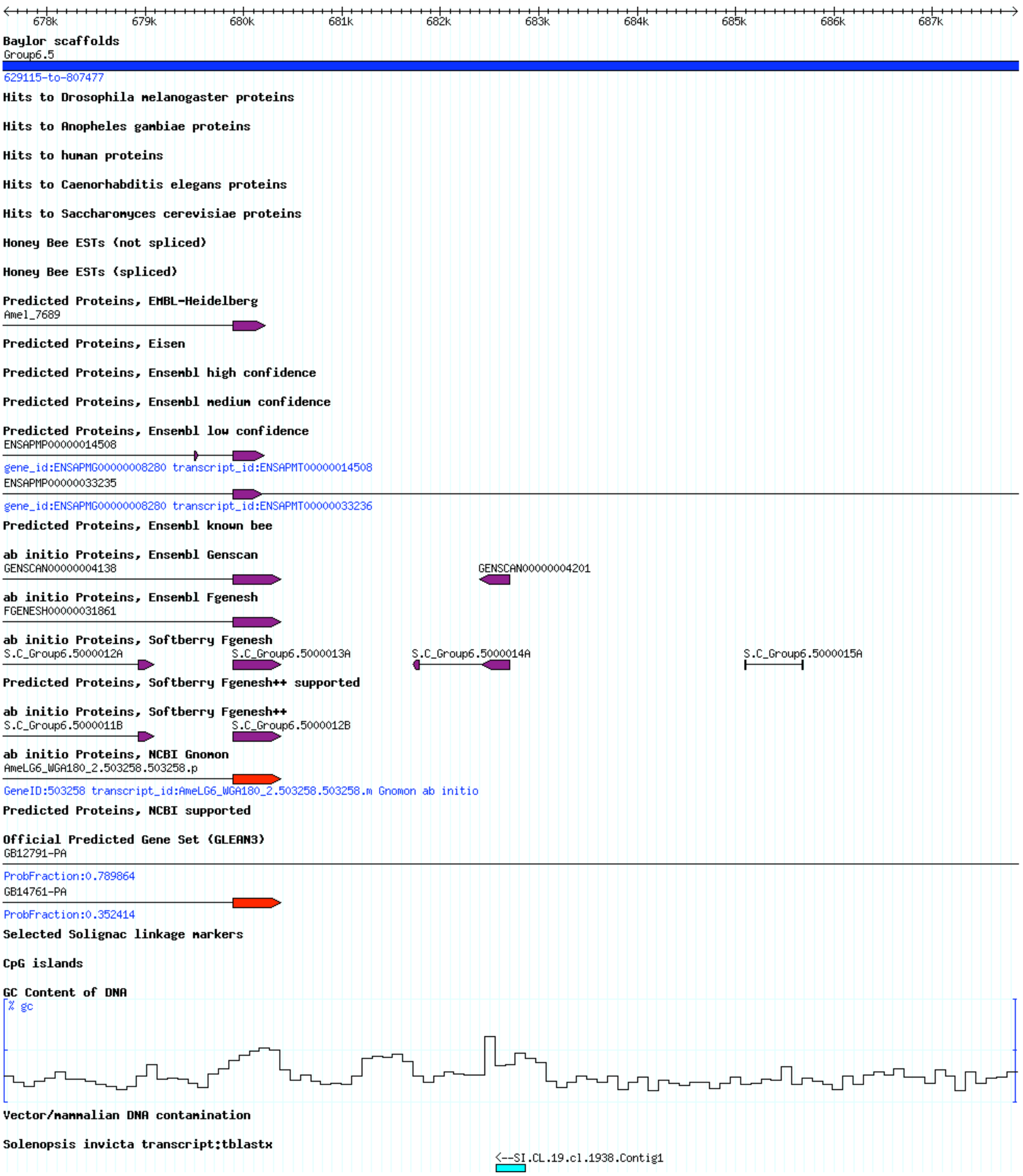

# i.SI.CL.19.cl.1953.SiJWC11BBX.scf

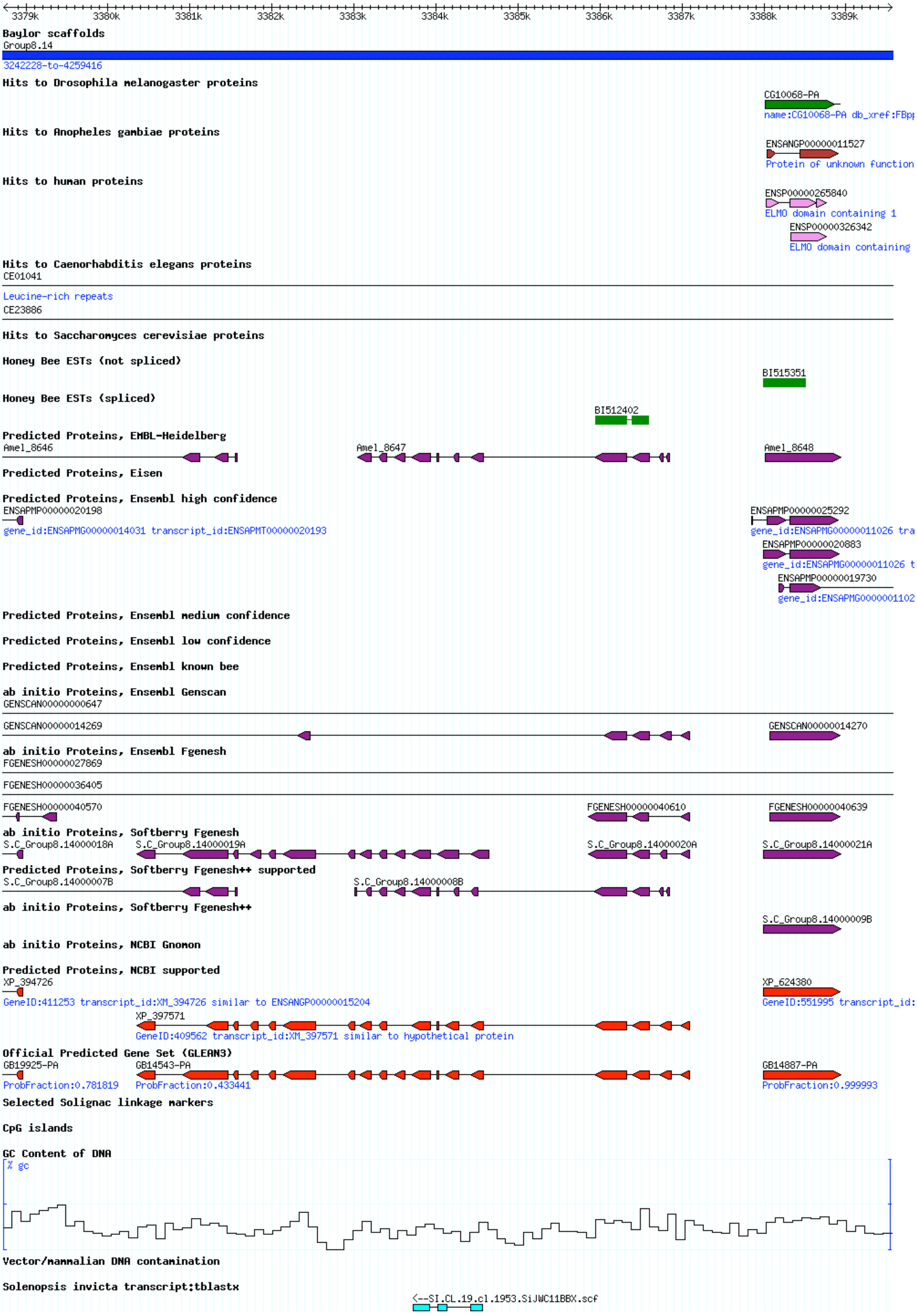

## j. SI.CL.23.cl.2326.Contig1

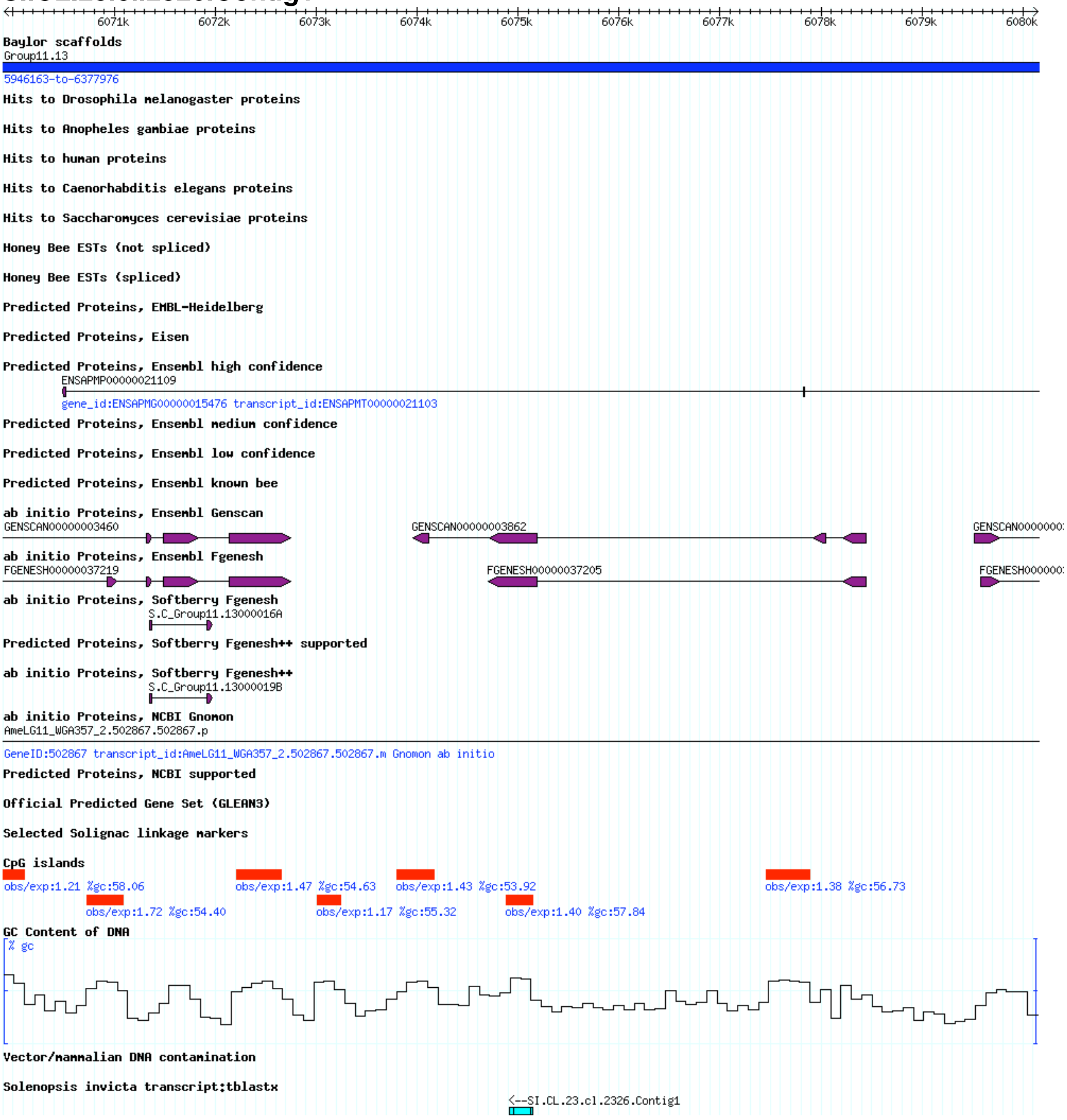

## k. SI.CL.26.cl.2688.Contig1

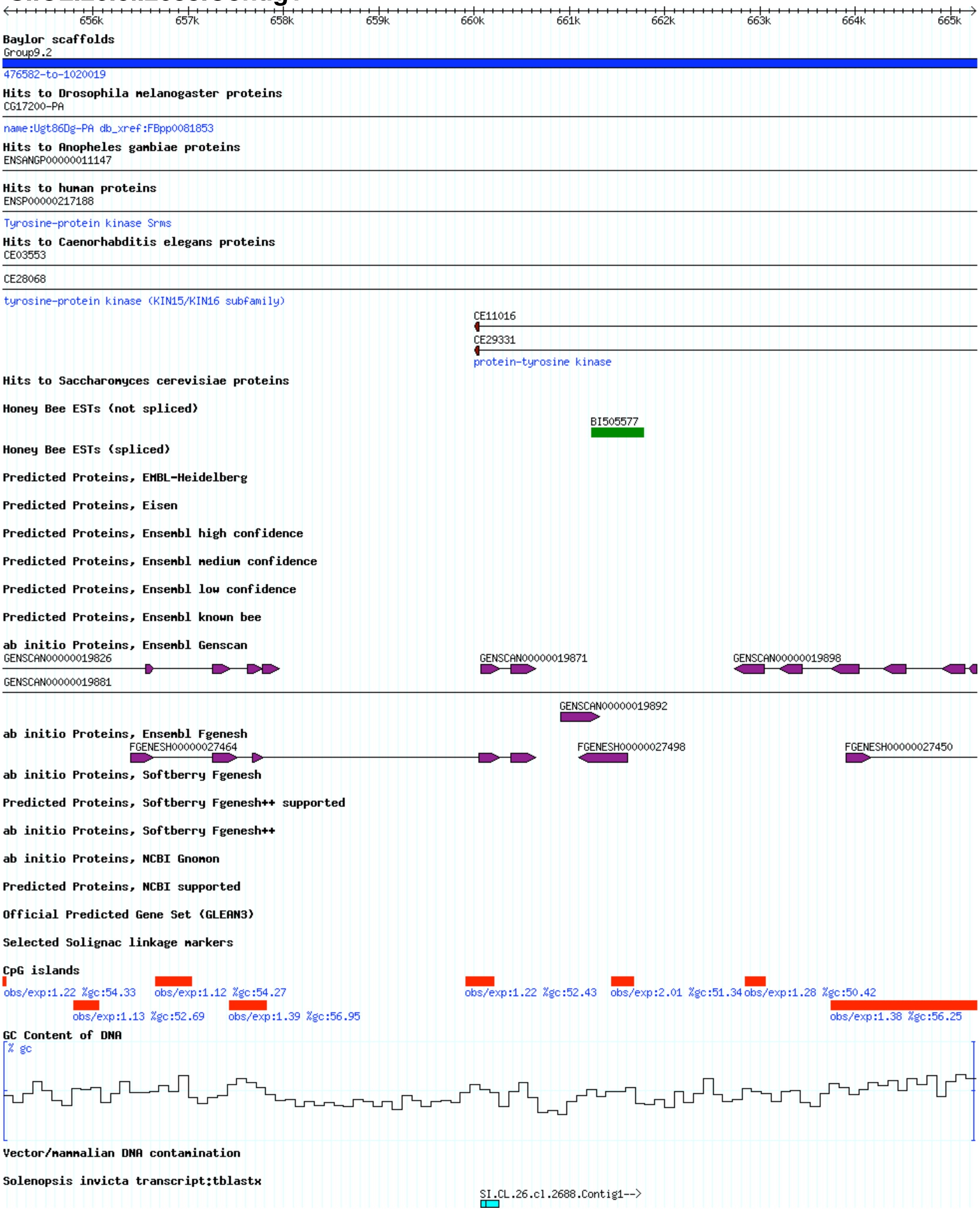

# I. SI.CL.33.cl.3311.Contig1

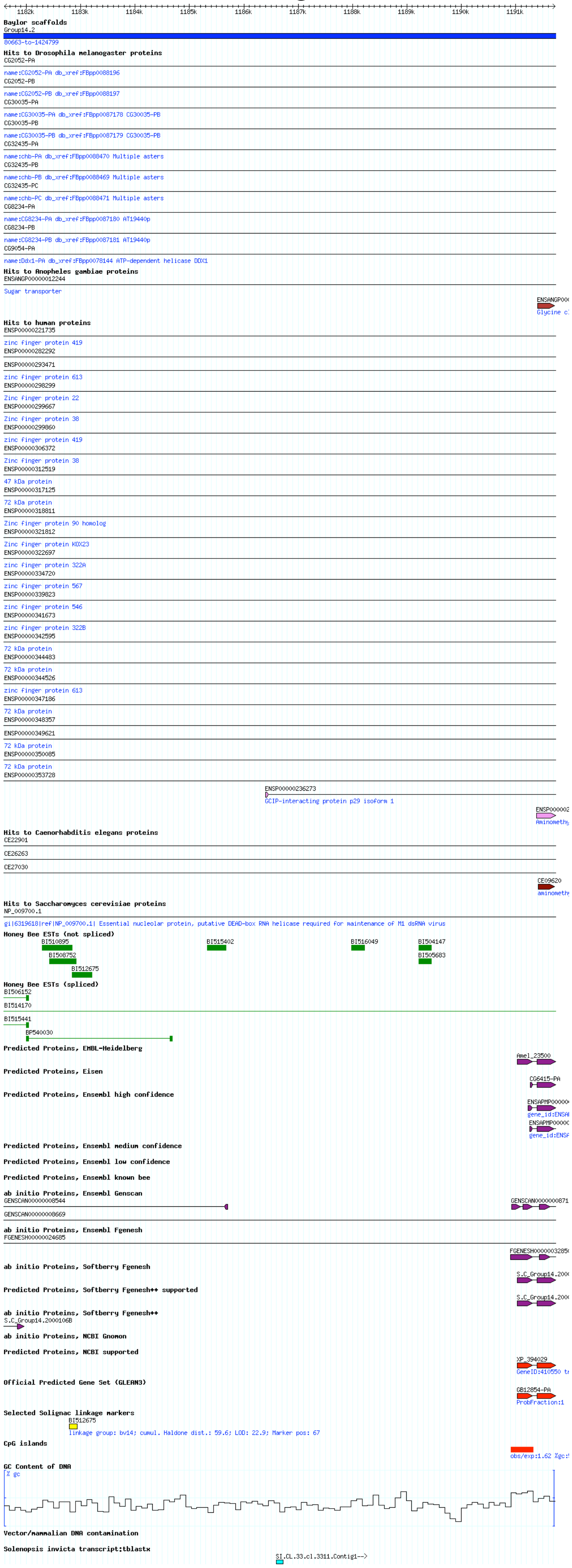

## m. SI.CL.33.cl.3384.Contig1

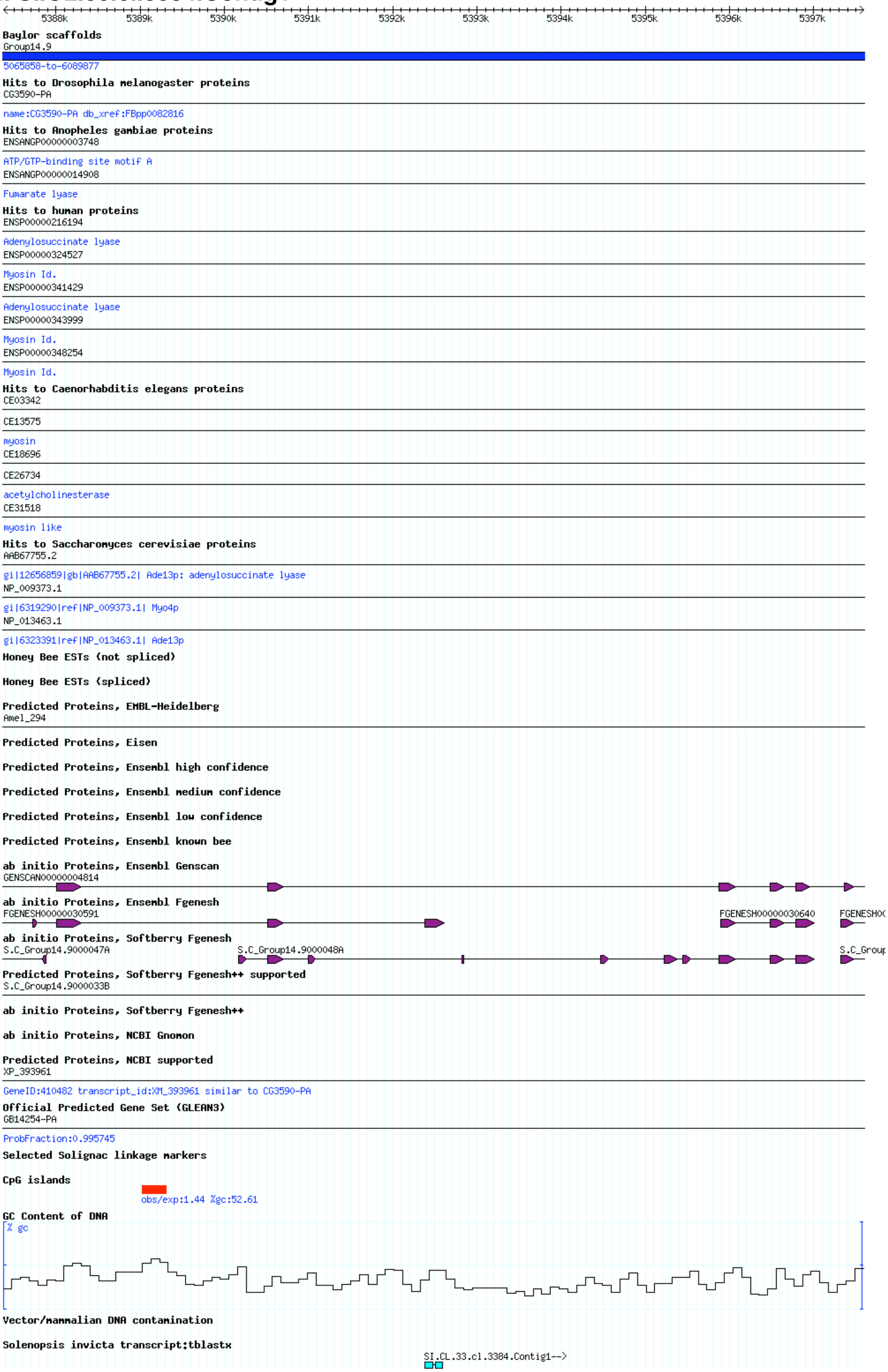

## n. SI.CL.35.cl.3595.Contig1

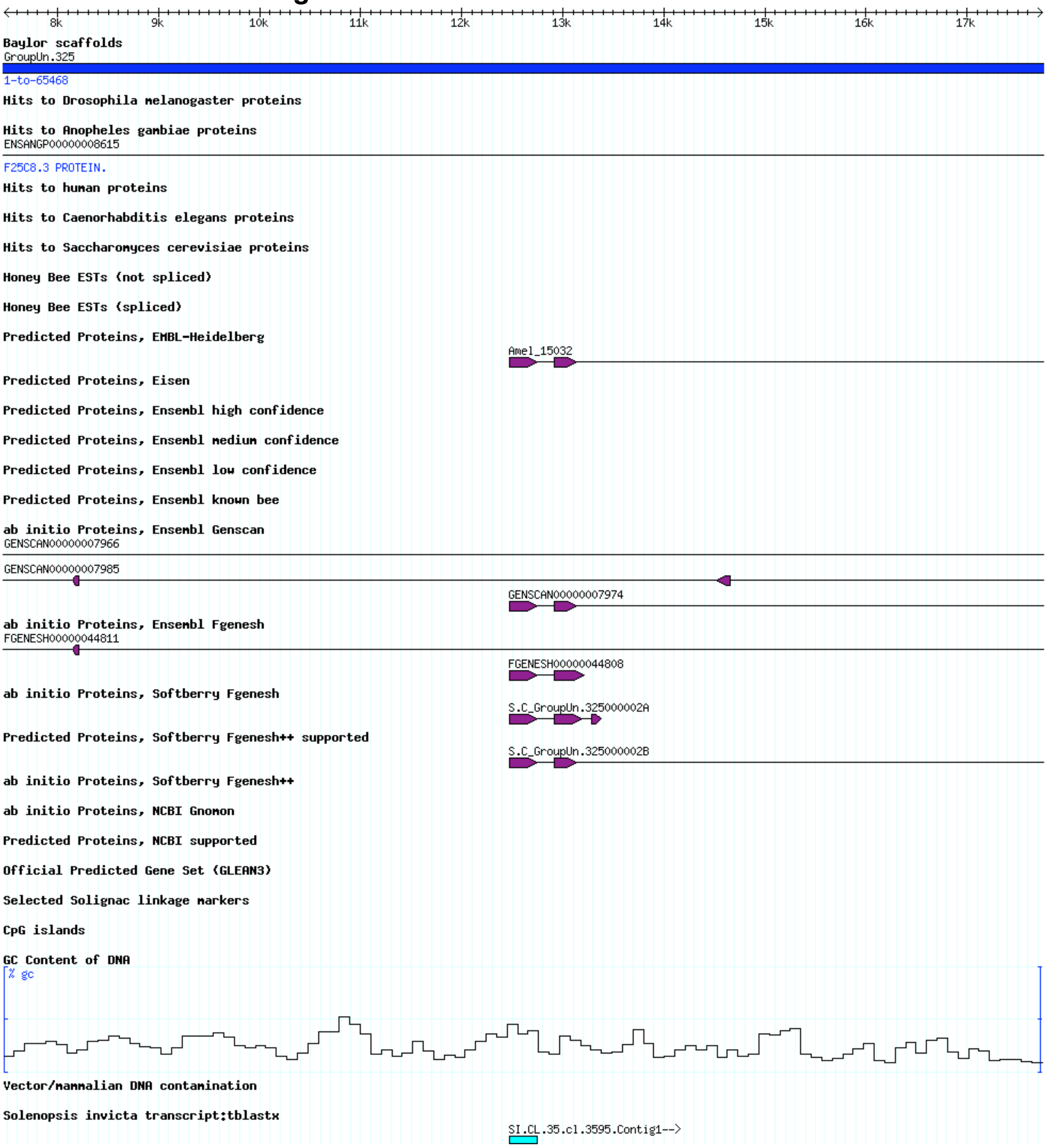

## o.SiJWE02BBO2.scf

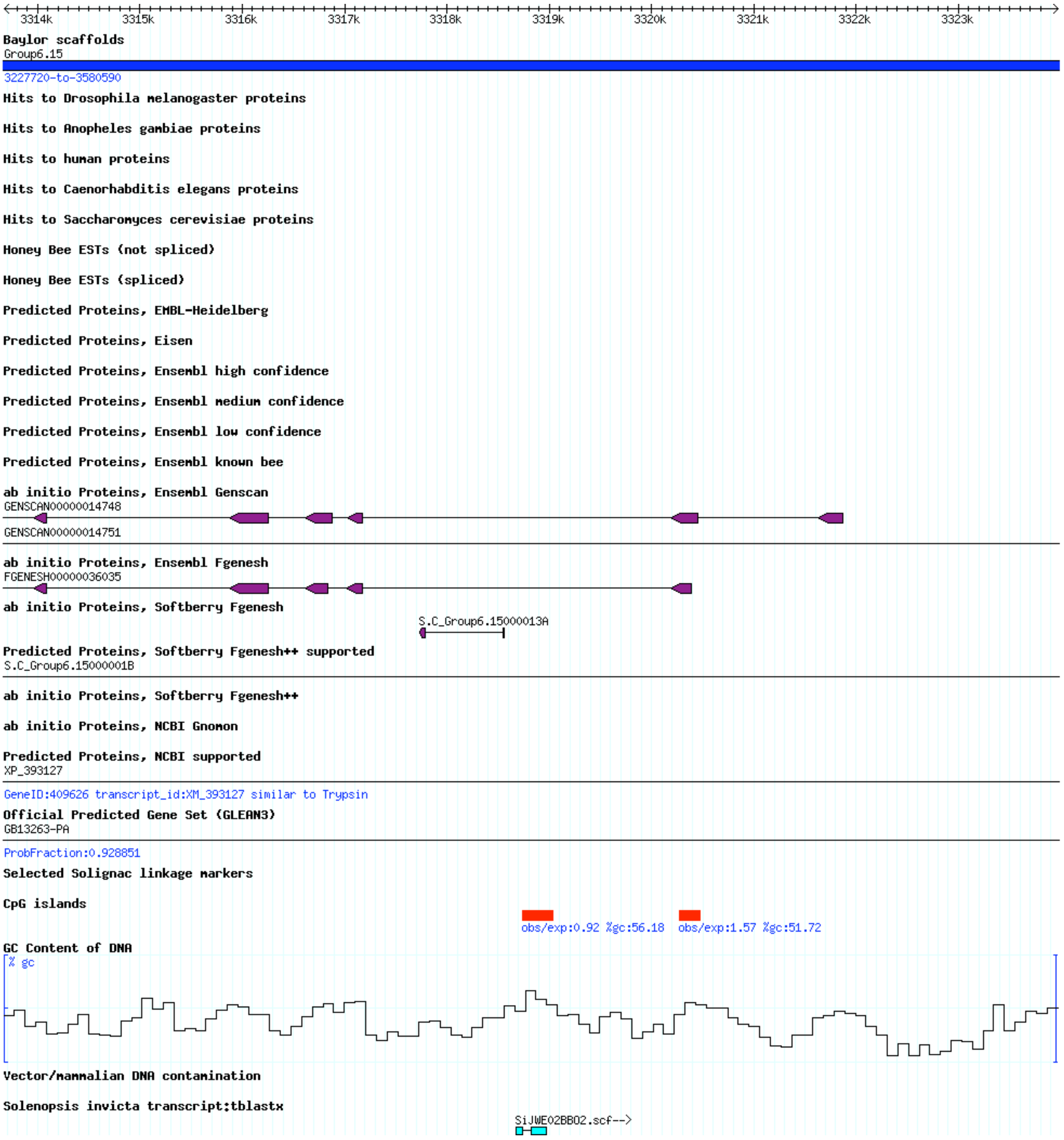

## p. SiJWF07BCC.tag5_F07_11.scf

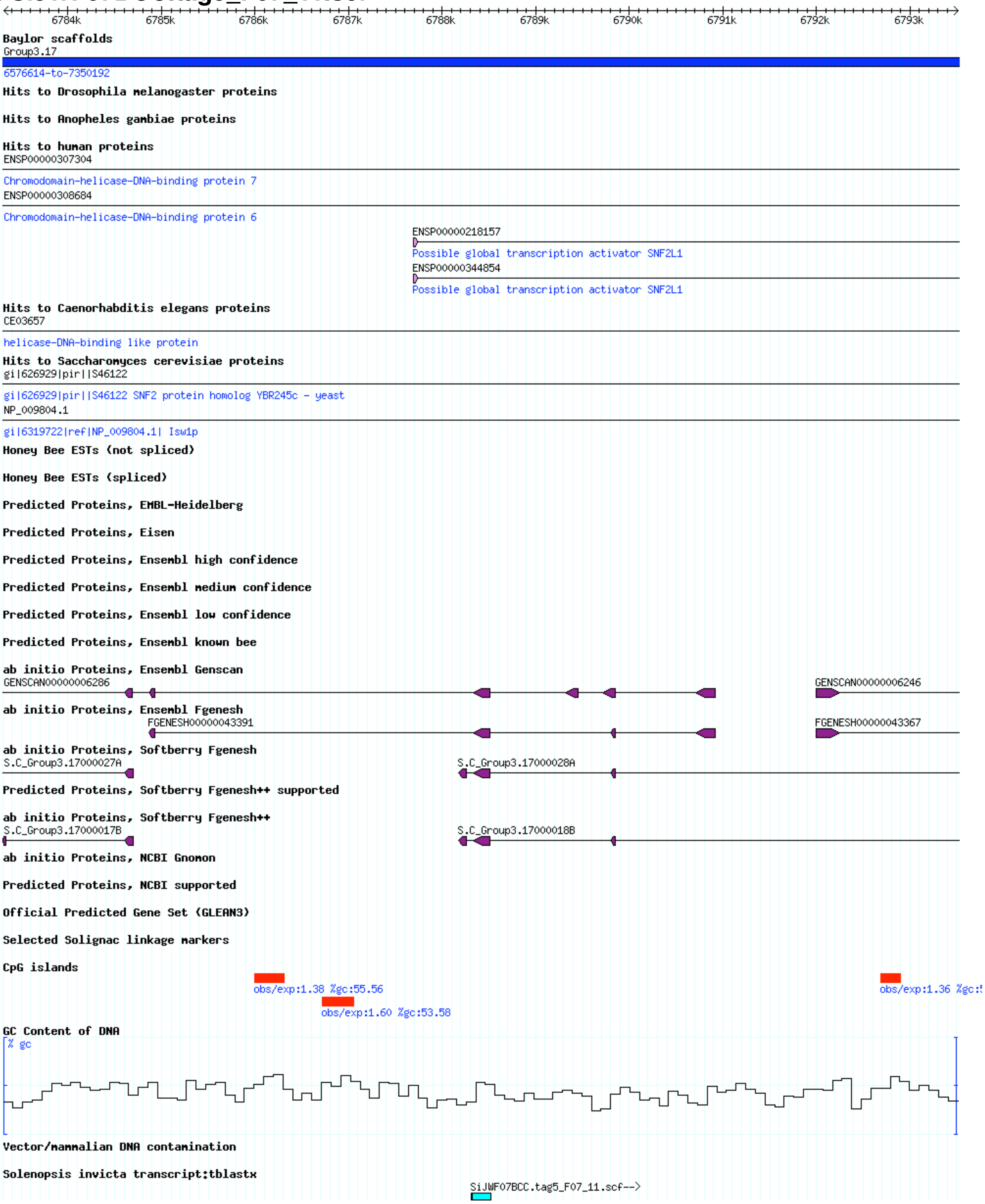

# q.SiJWG01BDU2.scf

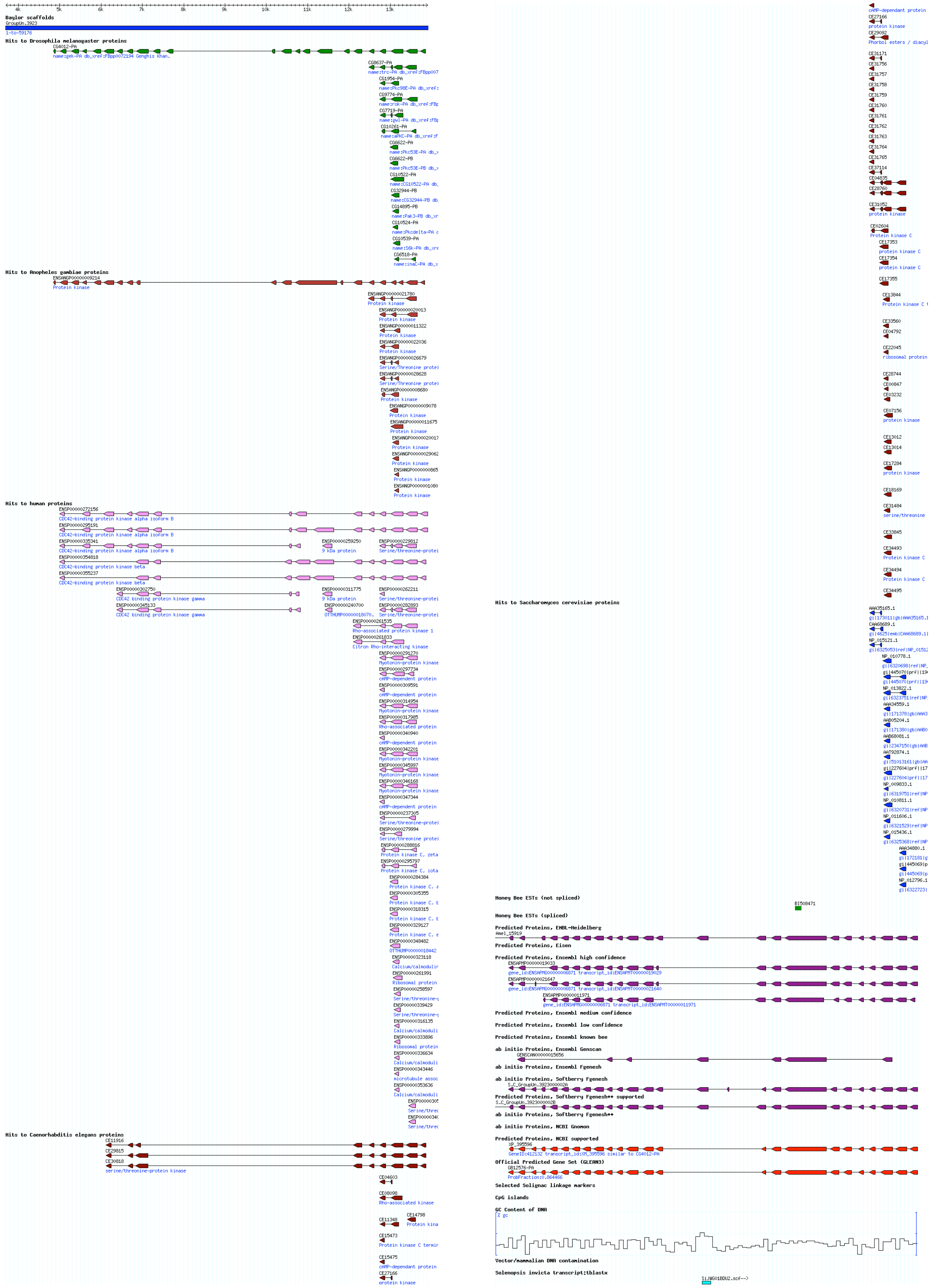

## r. SiJWG03ACB.scf

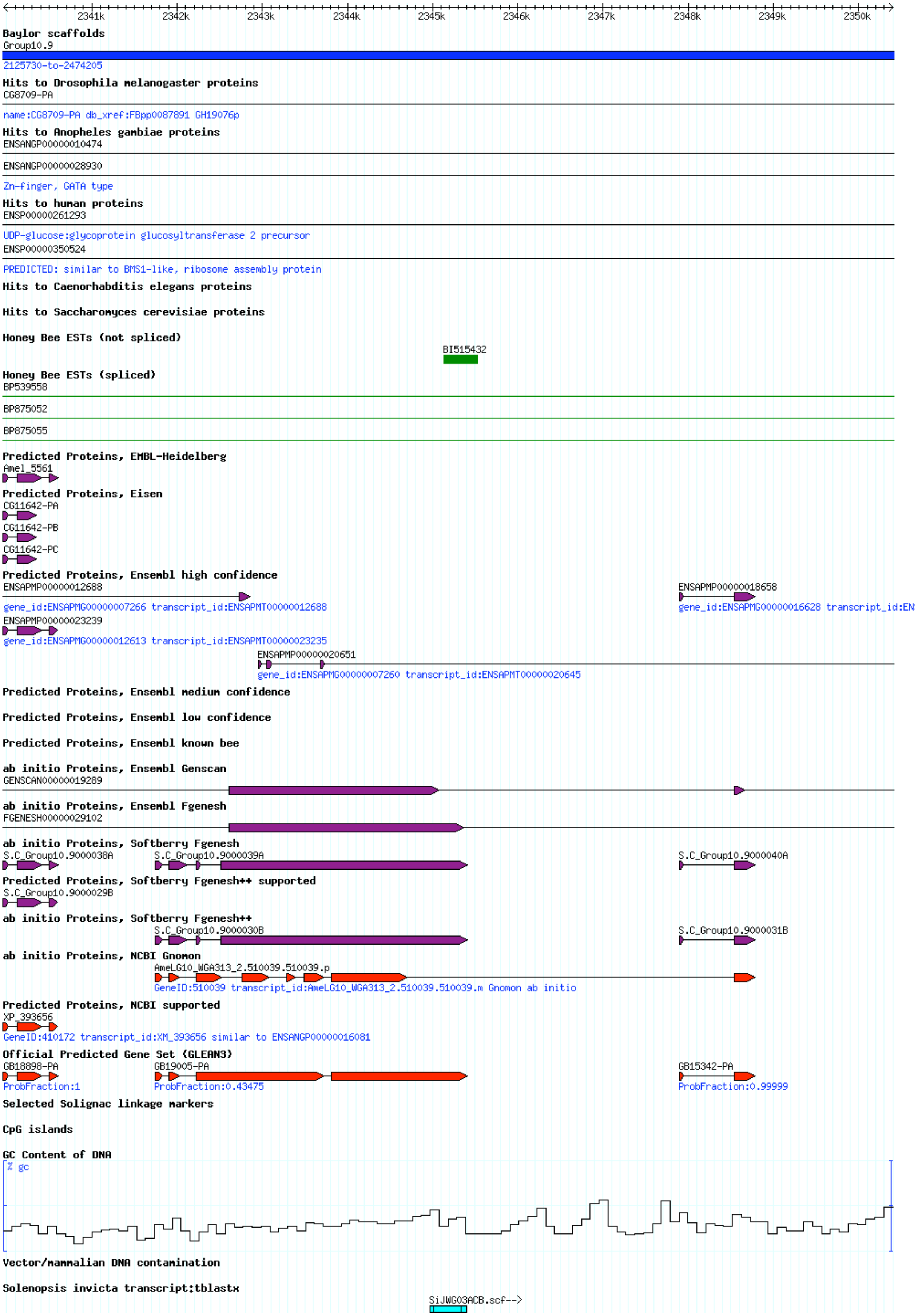

## s. SiJWH02AAN.scf

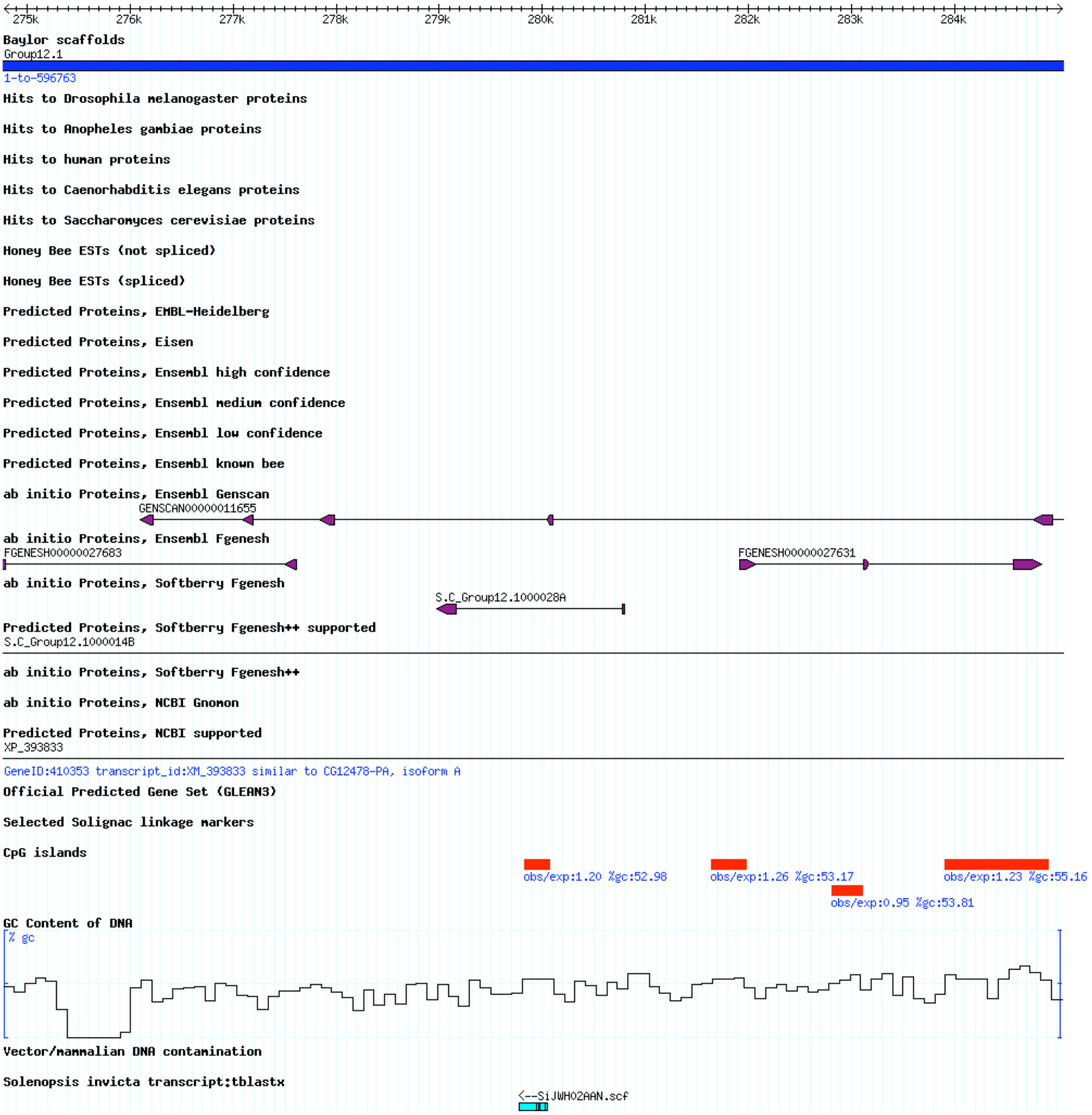

## t. SiJWH05BDPR5A08.scf

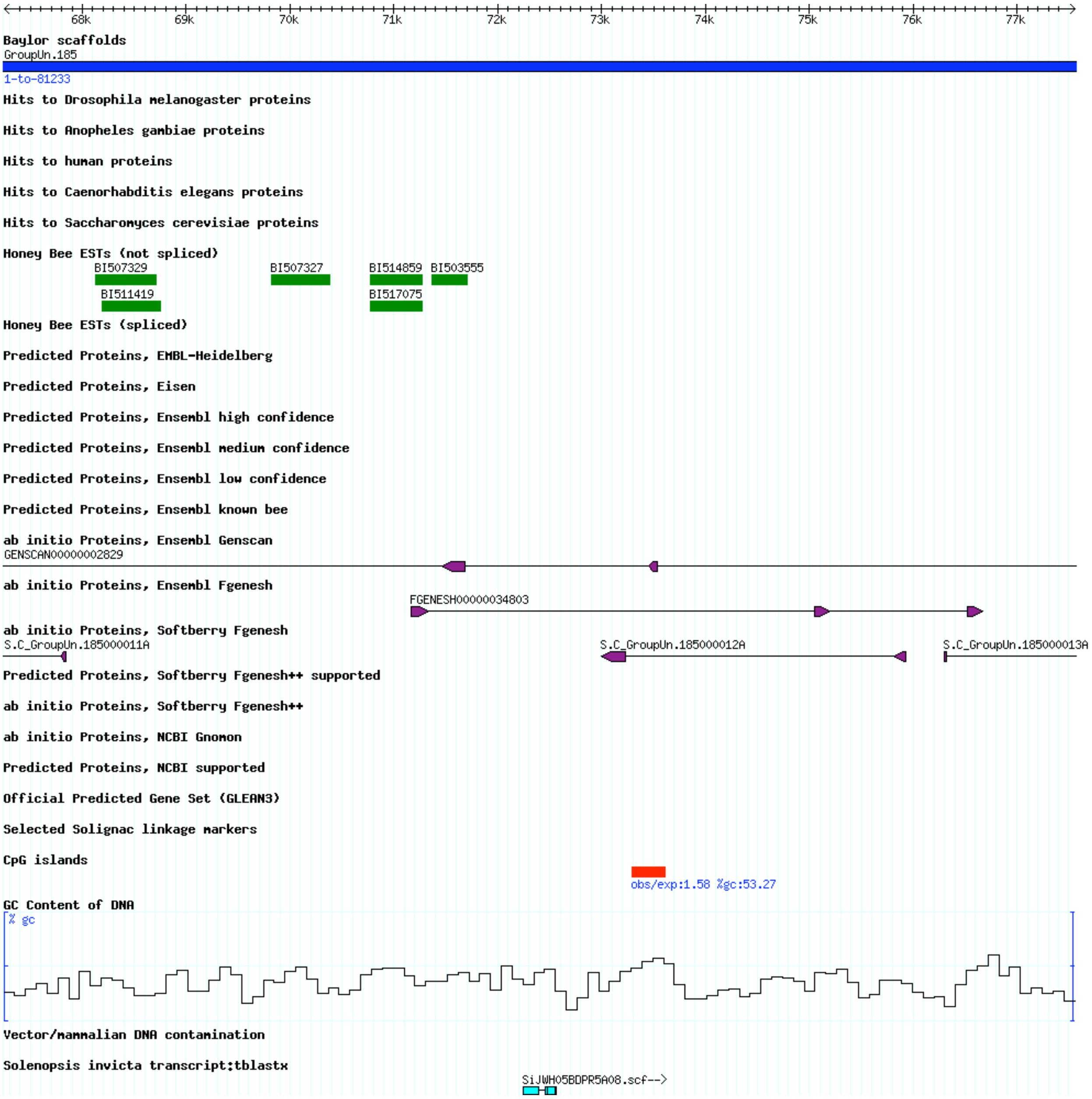

## u. SiJWH05BDV2.scf

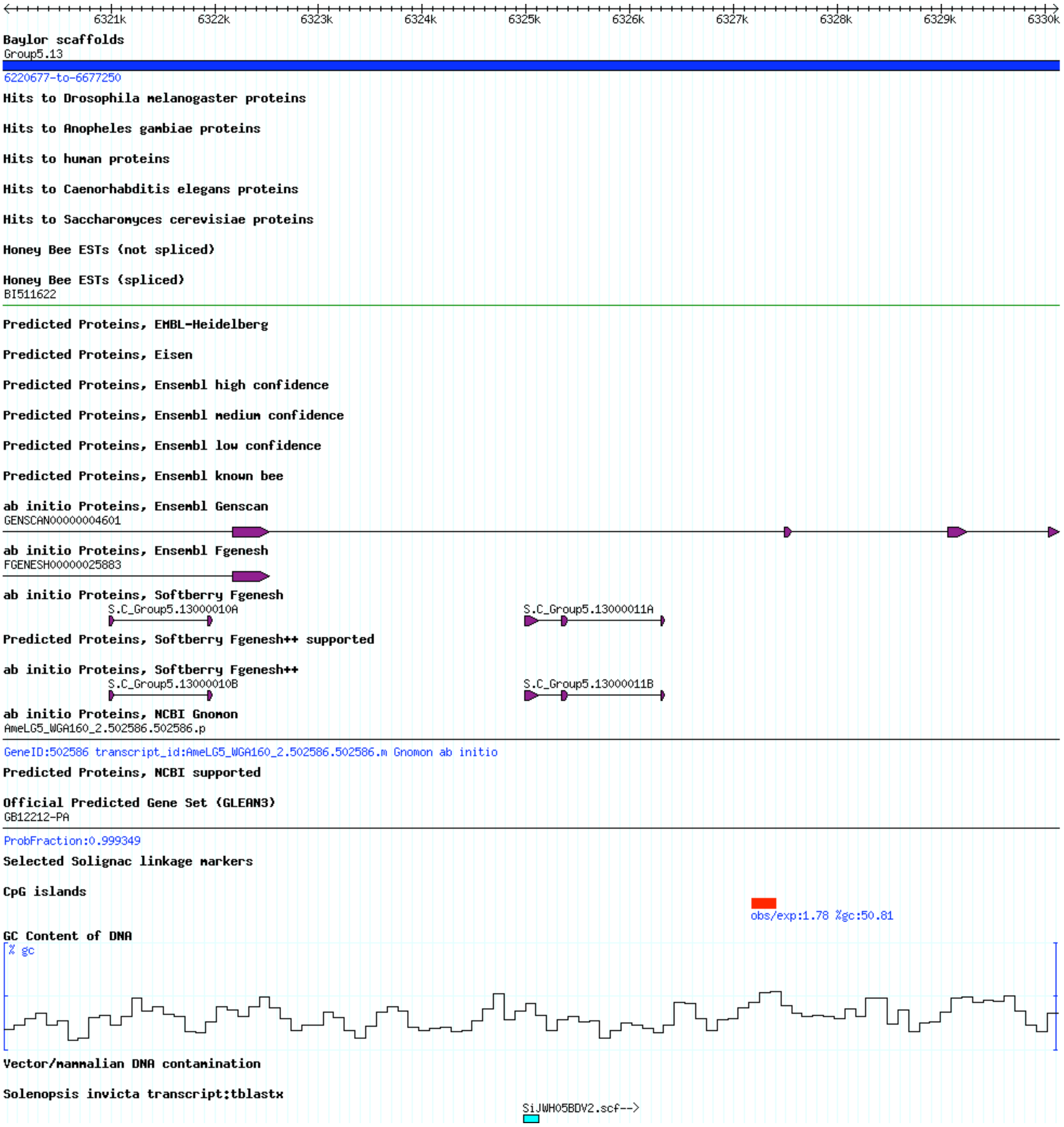

# v.SiJWH08AAT.scf

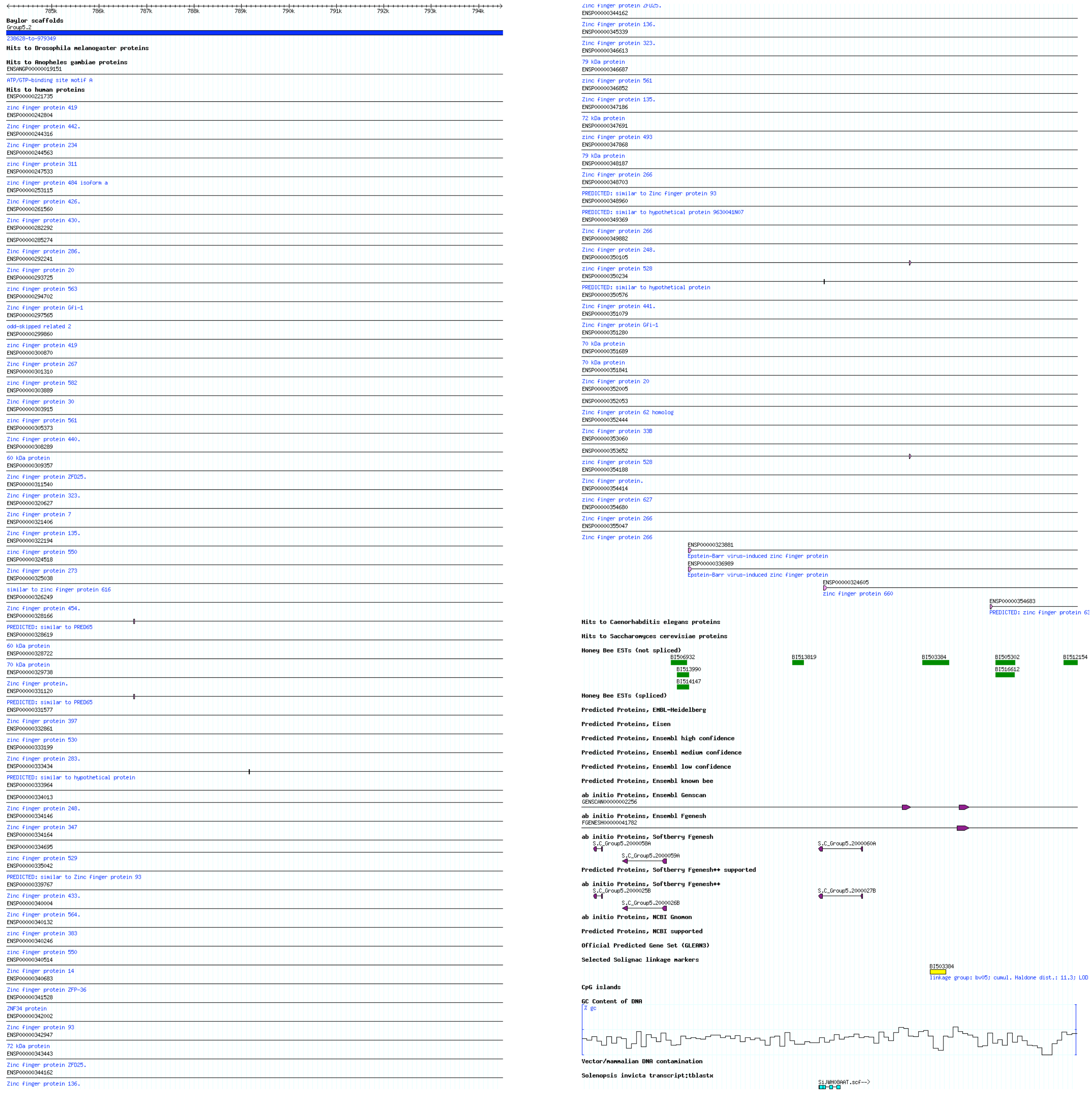

# w.SiJWH08ADY

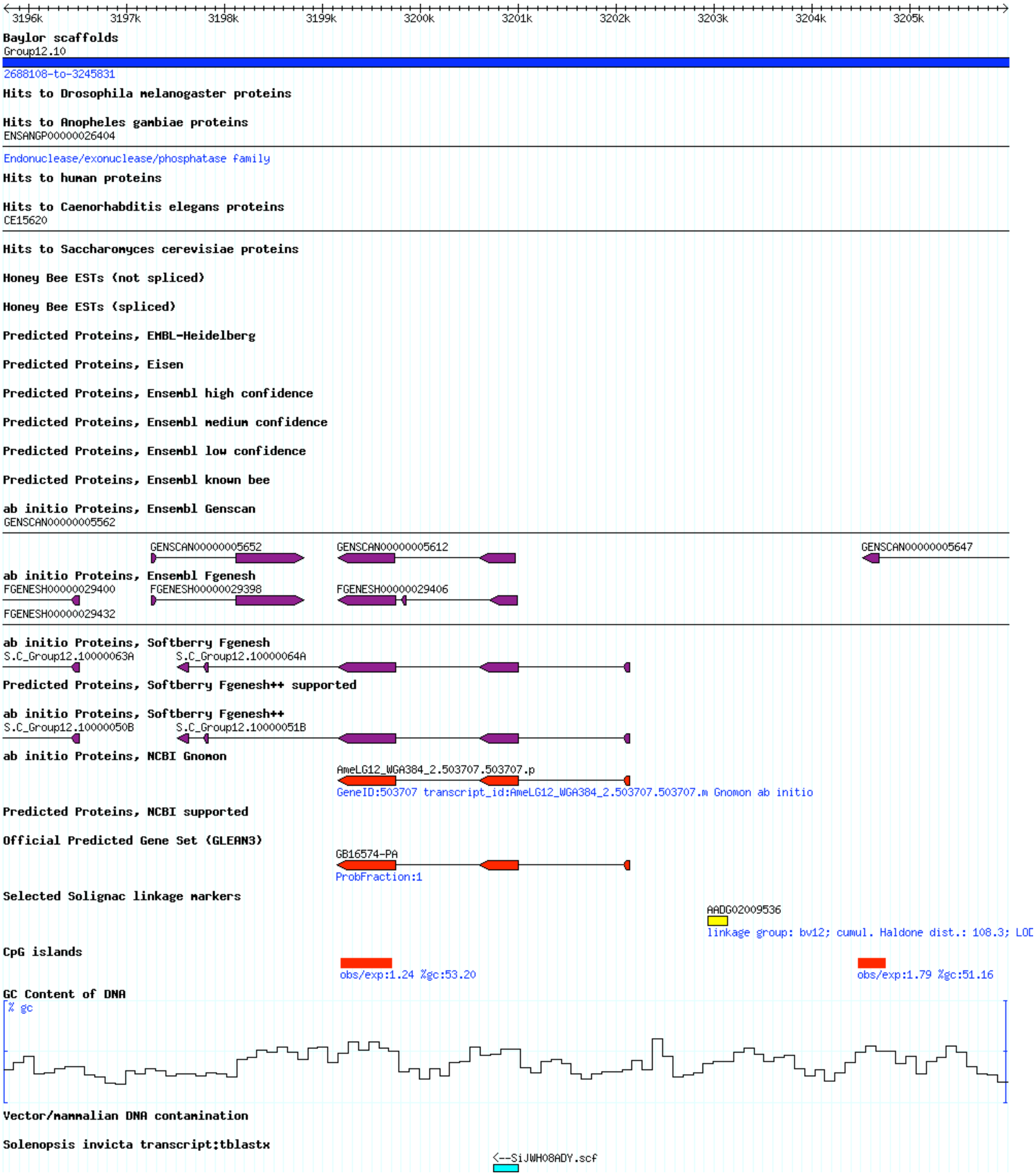

Supplement: Additional data file 5 — Honey bee genome regions surrounding the candidate Hymenoptera-specific genes listed in Table 3 [file gb-2007-8-1-r9-S5.pdf]
