# Supplementary material for: An annotated cDNA library and microarray for large-scale gene-expression studies in the ant Solenopsis invicta
Source: Genome Biol. 2007 Jan 15;8(1):R9. doi: 10.1186/gb-2007-8-1-r9 (PMC1839134; doi:10.1186/gb-2007-8-1-r9)
Supplement: Additional data file 10 — Details on the microarray analyses performed [file gb-2007-8-1-r9-S10.pdf]

Additional data file 10

Evaluation of the fire ant microarray

To evaluate the percentage of cDNA spots derived from legitimate and sufficiently highly expressed transcripts, we examined the signal-to-background value of all spots in four test hybridizations. These four hybridizations were technical replicates of two biological samples.

Biological Samples:

Brood: eggs, larvae and pupae of all castes in equal amounts

Adults: workers, virgin queens, and males from both colony types in equal amounts

Total RNA was amplified using the MessageAmp II kit (Ambion). “Brood” RNA was independently amplified three times while “Adult” RNA was independently amplified two times. Amplified RNA was labeled using the “indirect” method where reverse transcription was performed in the presence of amino-allyl dUTP and the resulting cDNA subsequently coupled to Cy3 or Cy5 fluorescent monomers. The samples were labeled and hybridized onto the microarrays as outlined below.

For each microarray, we counted the number of spots that were derived from a single good PCR product and that had a signal intensity greater than background plus two standard deviations for either the Cy3 or Cy5 labeled samples. Using the conservative criterion that a spot has sufficiently intense signal on all four microarrays, 82.8% (14,642/17,685) were satisfactory. On average 93.8% (16,588/17,685) had an adequate signal. These data indicate that most cDNA clones are derived from legitimate transcripts.

Supplementary Table Microarray: Hybridization schematic and the number of spots with satisfactory signal for each microarray

	Sample*		
Slide ID	Cy3-labeled	Cy5-labeled	Spots with adequate signal
B109	adult-1	brood-1	16,203
B110	brood-2	adult-1	16,534
C021	adult-2	brood-3	16,794
C022	brood-3	adult-2	16,822
Total spots on microarray (1 pcr product)			17,685
Average of 4 microarrays			16,588.25 (93.8%)
All hybridizations			14,642 (82.8%)

* Numbers refer to different amplifications from same initial total RNA

Adult and Brood enriched genes

We generated lists of potentially adult- or brood-enriched genes using two methods. In the first method we used a simple 4-fold expression difference cutoff (Additional data file 11). In the second method we performed a 1-sample t-test using a p-value cutoff of 0.001 (Additional data file 12). For both analyses, data were first normalized (loess normalization by print-tip group) using the marray package of Bioconductor (R).

We have not performed additional statistical analyses on this data set as these microarray data are derived from single biological samples.
